# Before They Ask: Anticipating Help-Seeking from Behavioral Signals in Virtual Reality

**DOI:** 10.3390/s26206361

**Published:** 2026-10-09

**Authors:** Sepideh Habibiabad, Jonas De Bruyne, Samuel Dossche, Pieter Simoens, Jelle Saldien, Klaas Bombeke

**Affiliations:** 1 imec-mict-UGent, Department of Communication Sciences, Ghent University, Miriam Makebaplein 1, 9000 Ghent, Belgium; jonas.debruyne@ugent.be (J.D.B.); samuel.dossche@ugent.be (S.D.); jelle.saldien@ugent.be (J.S.); klaas.bombeke@ugent.be (K.B.); 2 Department of Industrial Systems Engineering and Product Design, Ghent University, Technologiepark-Zwijnaarde 46, 9052 Ghent, Belgium; 3 Department of General and Applied Psychology, Ghent University, Henri Dunantlaan 2, 9000 Ghent, Belgium; 4 IDLab-imec, Department of Information Technology, Ghent University, Technologiepark 126, 9052 Ghent, Belgium; pieter.simoens@ugent.be; 5 imec-mict-UA, Department of Product Development, University of Antwerp, Paardenmarkt 94, 2000 Antwerp, Belgium

**Keywords:** virtual reality, help-seeking, self-regulated learning, intelligent tutoring systems, multimodal learning analytics

## Abstract

Immersive virtual reality (VR) is increasingly used for training and has the ability to track learner behavior continuously. Yet support is usually delivered only once a learner asks for it, and help is not always sought when needed. While support based on performance is common, less is known about detecting an emerging need for help from non-verbal behavior. We examined whether an upcoming help request can be anticipated from movement and gaze alone. We analyzed data from 21 participants who completed a VR assembly task in which rules changed without warning and could be consulted through a deliberate, logged action. All models were evaluated on unseen participants. Trial-level prediction was accurate but relied mainly on task outcomes, such as errors and earlier consultations, rather than on the learner’s ongoing behavior. Using short, continuously updated windows of movement and gaze, the model reached an AUC-PR of 0.159 against a chance level of 0.064, about 2.5 times the chance level, and most of its discriminative ability remained when the final turn toward the source of help was excluded. Before a request, movement slowed, gaze stayed closer to the head’s forward direction, and fixations lengthened. Help-seeking is thus partly foreshadowed by behavior VR systems already record, though the signal is still weak when generalized to new learners, who varied substantially in how often they sought help.

## 1. Introduction

Virtual reality (VR) is increasingly adopted for education and training because it enables realistic and interactive learning experiences that would otherwise be unsafe, expensive, or impractical to reproduce. Meta-analyses have shown that VR can improve learning outcomes, with benefits reported particularly for procedural and spatial learning, alongside gains in engagement and motivation [1,2]. VR also allows learners to practice repeatedly under controlled and consistent conditions. These advantages have led to successful applications across many domains. In medical and nursing education, VR is used to let students rehearse clinical procedures without risk to patients [3]. In engineering education, immersive virtual laboratories provide access to complex equipment and three-dimensional systems that are otherwise costly or difficult to access [4]. In high-risk domains such as construction, VR enables workers to practice recognizing and responding to hazards without exposure to dangerous real environments [5].

Beyond its educational benefits, VR offers a unique opportunity to study learner behavior. Modern VR systems continuously record rich multimodal behavioral data, including head and hand movements, eye gaze, and interactions with the virtual environment [6,7]. These signals can be time-stamped and synchronized with events occurring within the virtual environment, providing a continuous record of learner behavior without additional sensors or interruptions to the learning process. Previous studies have used such behavioral signals to investigate learner states and behavior in immersive learning environments [8,9]. VR is therefore not only a setting for training, but also a natural environment for observing learner behavior as it unfolds.

This continuous behavioral sensing also creates opportunities for personalized learning, in which instruction is adapted to the individual rather than delivered uniformly to all learners. Learners differ in prior knowledge, learning pace, and the concepts they find difficult; under one-size-fits-all instruction, struggling learners risk cognitive overload while more advanced learners may disengage [10]. Personalized instruction can adapt task difficulty and support to keep learners within their “zone of proximal development”, where challenging tasks become achievable with appropriate guidance [11,12]. Flow theory offers a complementary perspective: engagement is greatest when challenge and skill are appropriately balanced, whereas excessive challenge can produce anxiety and insufficient challenge can lead to boredom [13]. Because this balance differs across learners, maintaining it requires individual adaptation, and flow during learning has been positively related to subsequent performance [14]. Consistent with this rationale, a meta-analysis of 34 studies reported a medium positive effect of technology-facilitated personalized learning on achievement [15], while a systematic review of 53 studies reported positive effects of intelligent tutoring systems across a range of educational domains [16].

The closest established model of this kind of individualized attention is one-to-one human instruction. Part of what makes it effective is that the tutor can observe the learner while they work and act on what is observed: human tutors infer learners’ states from the ongoing interaction and adjust their subsequent moves accordingly [17]. Providing this level of attention at scale, however, is difficult. In classroom settings, instructors must divide limited time and attention across many learners working on divergent activities, making it difficult to continuously monitor any individual learner’s problem-solving process [18]. Intelligent tutoring systems offer a scalable complement to human instruction. Evidence suggests they can approach human tutoring in learning outcomes: a review of tutoring experiments found intelligent tutoring systems and human tutors similarly effective relative to no-tutoring controls [19], and a meta-analysis found no significant difference between intelligent tutoring systems and individualized human tutoring [20]. A remaining challenge for such systems is inferring from a learner’s ongoing behavior when support is needed.

Intelligent tutoring systems can maintain a model of the learner based on interaction and performance data [16]. This learner model can be updated as the learner progresses through a task and used to adapt the support provided [21]. Beyond evaluating whether an answer is correct, some tutoring systems can estimate aspects of the learner’s cognitive and affective state, such as confusion, and adapt their guidance accordingly [22,23]. This creates the possibility of responding to difficulties as they emerge rather than only after a task outcome is observed.

The timing of support matters because intervening too early and too late can both carry costs. Allowing learners to first engage with a difficult problem, including unsuccessful attempts, can support deeper learning from subsequent instruction [24,25]. Encountering and working through a difficulty can itself contribute to learning [19]. Conversely, when difficulty remains unresolved, productive effort may give way to frustration, disengagement, or boredom [26,27]. This trade-off is captured by the assistance dilemma: a tutor must determine not only what support to provide, but also when and how much [28]. Cognitive load theory adds a complementary constraint, as insufficient guidance during complex tasks can overload working memory [29]. Consistent with the importance of appropriately timed guidance, delaying visual guidance in a procedural VR training task has been shown to improve procedural learning without increasing cognitive load [30].

Effective support therefore depends not only on whether help is provided, but also on when. This timing problem is particularly challenging in self-paced digital environments, where learners may encounter difficulty without explicitly requesting assistance. Indeed, learners sometimes fail to seek help when it is needed, and such help avoidance has been associated with poorer learning outcomes [31]. This makes help-seeking particularly relevant to the timing problem: anticipating an upcoming request from the learner’s ongoing behavior could allow a tutor to respond before the request itself occurs.

Help-seeking, the behavior we target, is commonly conceptualized as a self-regulated learning strategy rather than simply a sign of dependence. Learners may seek help deliberately to overcome difficulty and continue working independently [32,33]. Because help-seeking follows from the learner’s monitoring of their own understanding, an explicit request provides an observable indication that the learner has recognized a difficulty or gap in their understanding [34]. Importantly, help-seeking is not a single act but a process. Nelson-Le Gall describes five stages: (1) becoming aware of a need for help through metacognitive monitoring, (2) deciding whether to seek help, (3) identifying an appropriate source of assistance, (4) employing strategies to elicit help, and (5) evaluating the outcome [32,35]. The explicit request therefore represents a later observable point in a process that begins earlier. If these preceding stages are reflected in observable behavior, they may leave detectable traces before the learner explicitly asks for help.

Tutoring systems can operate reactively, providing feedback or guidance in response to learner input, such as an answer, error, or explicit request for help [36,37]. When assistance depends on an explicit request, however, learners who avoid seeking help may not receive support when they need it [31]. More recent intelligent tutoring systems increasingly incorporate ongoing learner monitoring and real-time adaptation [38], creating the possibility of responding before an explicit request occurs. In terms of the help-seeking process described above, this requires identifying behavioral signs that precede the learner’s overt attempt to elicit help.

Non-verbal behavior provides a potential source of information while the learner is actively engaged in a task, without requiring an explicit self-report. Such signals are not direct measures of a learner’s internal state, but prior work suggests that they can reflect cognitive and affective processes relevant to learning. In mixed reality environments, eye, head, and hand movement features have been associated with different levels of cognitive load [8]. Eye-movement measures have also been associated with decision uncertainty [39]. Upper-body and head movements have been shown to vary with task difficulty [40], while hand movement patterns differ with domain expertise and can change with problem difficulty [41]. Non-verbal cues have additionally been used to classify learners’ attentive and inattentive states [42]. Together, these findings suggest that non-verbal behavior can provide information about learner processes while interaction is ongoing, although they do not establish a direct relationship with help-seeking.

Beyond reflecting current learner processes, non-verbal behavior can also carry predictive information. In face-to-face interactions between teachers and students, automatically tracked body movements have been used to predict students’ subsequent test performance [43]. More directly relevant to anticipating behavior, a review of gaze-based intention estimation describes gaze as an early cue to subsequent action [44]. In VR, gaze dynamics have been used to predict the onset of item selection [45], and adding hand–eye coordination features further improved the recognition of interaction intention [46], although these predictions targeted horizons of well under one second. Gaze has also been used to infer when users need assistance: gaze sequences recorded during VR navigation were used to predict when users needed a navigation aid, so that the aid could be displayed only when needed [47], and eye-movement features have been used to identify when users need wayfinding assistance [48]. Beyond gaze, changes in head movement have been observed before pedestrians initiated a crossing decision in a virtual environment [49]. Although these studies address different tasks and contexts, they show that non-verbal signals carry information about users’ current needs and, in some cases, their upcoming actions.

Predictive models in intelligent tutoring and adaptive learning have targeted a range of future learner behaviors, states, and outcomes using behavioral and interaction data. Reading behavior on previous texts has been used to predict whether a learner will quit an upcoming text before beginning it [50], while knowledge-tracing models have incorporated simulated affective trajectories alongside knowledge states to improve prediction of future performance [51]. Closer to the present approach, predictions have also been made from continuously sampled physiological and oculomotor signals. Real-time pupil dilation has been used to identify low-workload moments and time task interruptions accordingly [52], while joint visual attention and mental-effort measures have been used to forecast suboptimal collaboration states between pair programmers up to 30 s in advance [53].

Within the narrower literature on help-seeking, several related approaches have been explored. In a logic-proof tutor, a data-driven model incorporating prior hint usage was used to predict when learners needed assistance, and proactively providing hints at these predicted moments reduced training time and improved post-test performance compared with a non-proactive control [54]. In a block-based programming environment, hint requests were clustered according to their timing and the completeness of the learner’s code, revealing distinct help-seeking patterns associated with prior knowledge and perceived skill [55]. More recently, problem-solving and help-seeking logs collected during the first minutes of a task have been used to identify learners’ help-seeking patterns and tendencies, with classifiers achieving early identification of these patterns [56]. These approaches rely primarily on interaction history and task-related behavior rather than continuously recorded non-verbal signals. Gaze-based work on anticipating a need for assistance in VR has so far focused on navigation [47,48]. To the best of our knowledge, anticipating the onset of a learner-initiated help request during a learning task from continuously recorded non-verbal behavior has not previously been investigated.

The present study addresses this gap using a rule-based assembly task in immersive VR. Participants completed 120 trials in which the governing rules changed without warning at three points during the session. The current rules could be consulted through a deliberate action directed toward a virtual instructor, providing precisely time-stamped help-seeking events (Section 2.3). Models were evaluated subject-independently so that performance reflected generalization to unseen learners. Prediction relied on eye, head, and hand movement recorded directly by the VR headset.

Using these signals, we address two research questions. First, can upcoming help-seeking be predicted from a learner’s non-verbal behavior before its onset (RQ1)? We examine this at two temporal resolutions: a trial-level analysis predicting whether help-seeking will occur in the following trial, and a window-level analysis predicting from short windows of continuous behavior whether a help-seeking episode will begin within the next few seconds. Second, exactly which non-verbal behaviors precede help-seeking and how do they change as a request approaches (RQ2)?

## 2. Materials and Methods

### 2.1. Participants

Twenty-two participants (8 male, 14 female; aged 24–46 years, M=30.7, SD=4.9) were recruited through convenience sampling, with no restrictions on prior VR experience. The only eligibility criterion was the absence of a color vision deficiency that would prevent distinguishing the four object colors used in the task; the color palette was nonetheless chosen to remain distinguishable under common forms of color vision deficiency. Before taking part, all participants received a written information letter and provided informed consent, and they were compensated for their time in accordance with institutional guidelines. The study protocol was approved by the Ethics Committee of the Faculty of Political and Social Sciences of Ghent University (reference number 2025-40), and the study was conducted in accordance with the committee’s guidelines. One participant was excluded from all analyses because of a failure in eye-tracking data collection, yielding a final sample of 21 participants.

### 2.2. Virtual Reality Environment

The experiment was conducted in a controlled laboratory setting. Participants stood freely in a cleared physical space to allow unrestricted movement throughout the session. The virtual environment was rendered using a Meta Quest Pro (Meta Platforms Technologies, LLC, Menlo Park, CA, USA) head-mounted display (HMD), which provides six-degrees-of-freedom (6-DoF) head tracking and integrated hand controller tracking. The task was developed in Unity 2021.3.45f1 using a built-in data logging plugin that handled trial sequencing, event time-stamping, and synchronized data recording across modalities.

**Figure 1:**
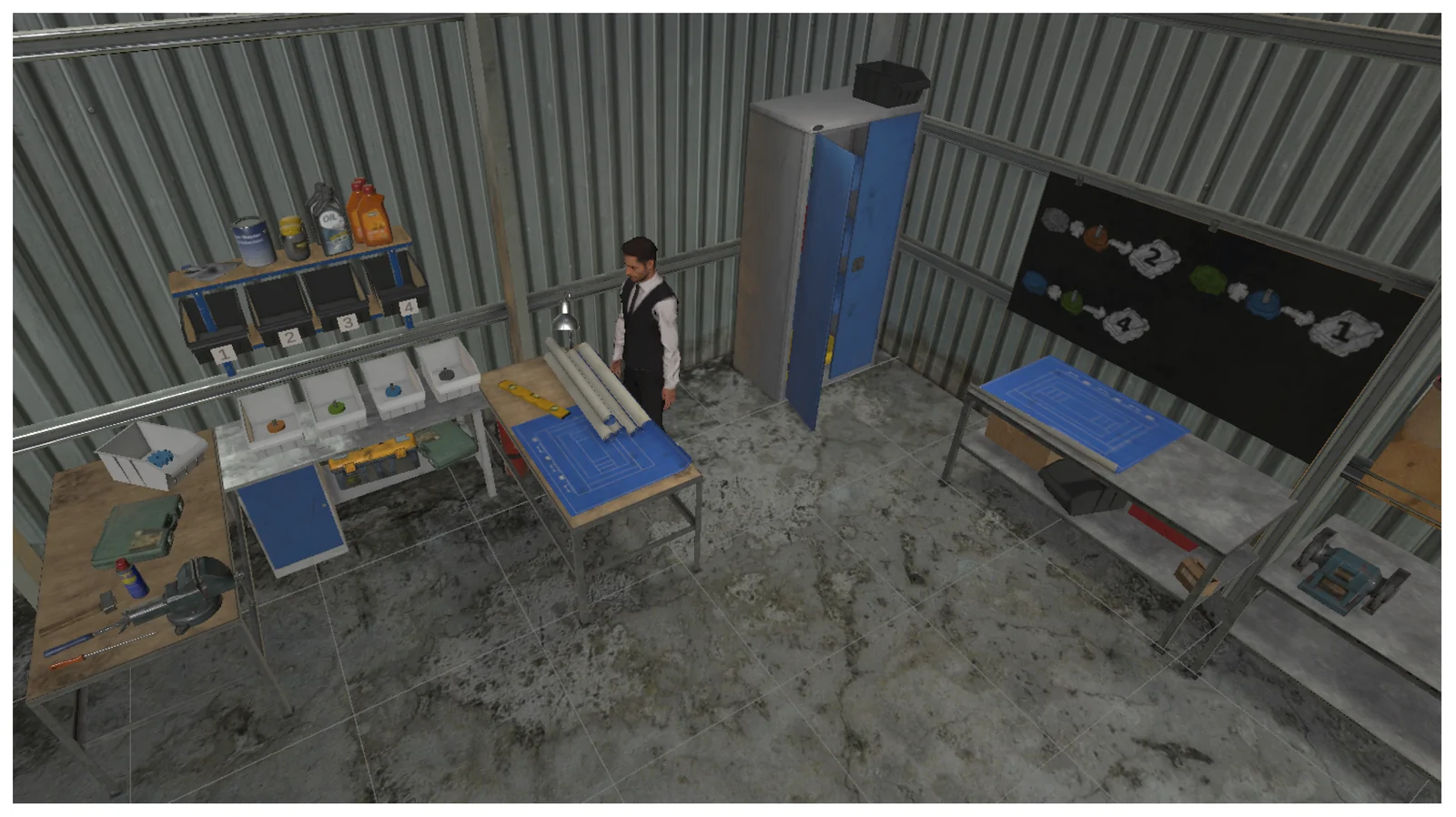
The virtual garage-style workshop environment used in the experiment. A gear is visible in the tray to the participant’s left; assembly bases are on the worktable in the foreground; numbered sorting bins are mounted on the wall above the worktable; the blackboard displaying assembly rules is on the right; the virtual avatar instructor stands in the center.

The virtual scene depicted a garage-style workshop (Figure 1). The environment included a worktable with four trays each holding a colored assembly base, a tray to the participant’s left holding the current trial’s gear, four numbered bins mounted on the wall directly above the worktable serving as sorting destinations, a blackboard displaying the current assembly rules, and a virtual avatar acting as an in-environment instructor. The avatar served two functions: before the experiment, it verbally guided participants through the task mechanics; during the experiment, it confirmed rule changes at a participant’s first help request after a rule change, as described in Section 2.3.

### 2.3. Task and Procedure

Before the main experiment, participants completed a self-paced introductory phase in which the avatar explained the environment, the objects, and the interaction mechanics. Participants could take as long as needed before indicating they were ready to begin. Data collection started with the first experimental trial.

**Figure 2:**
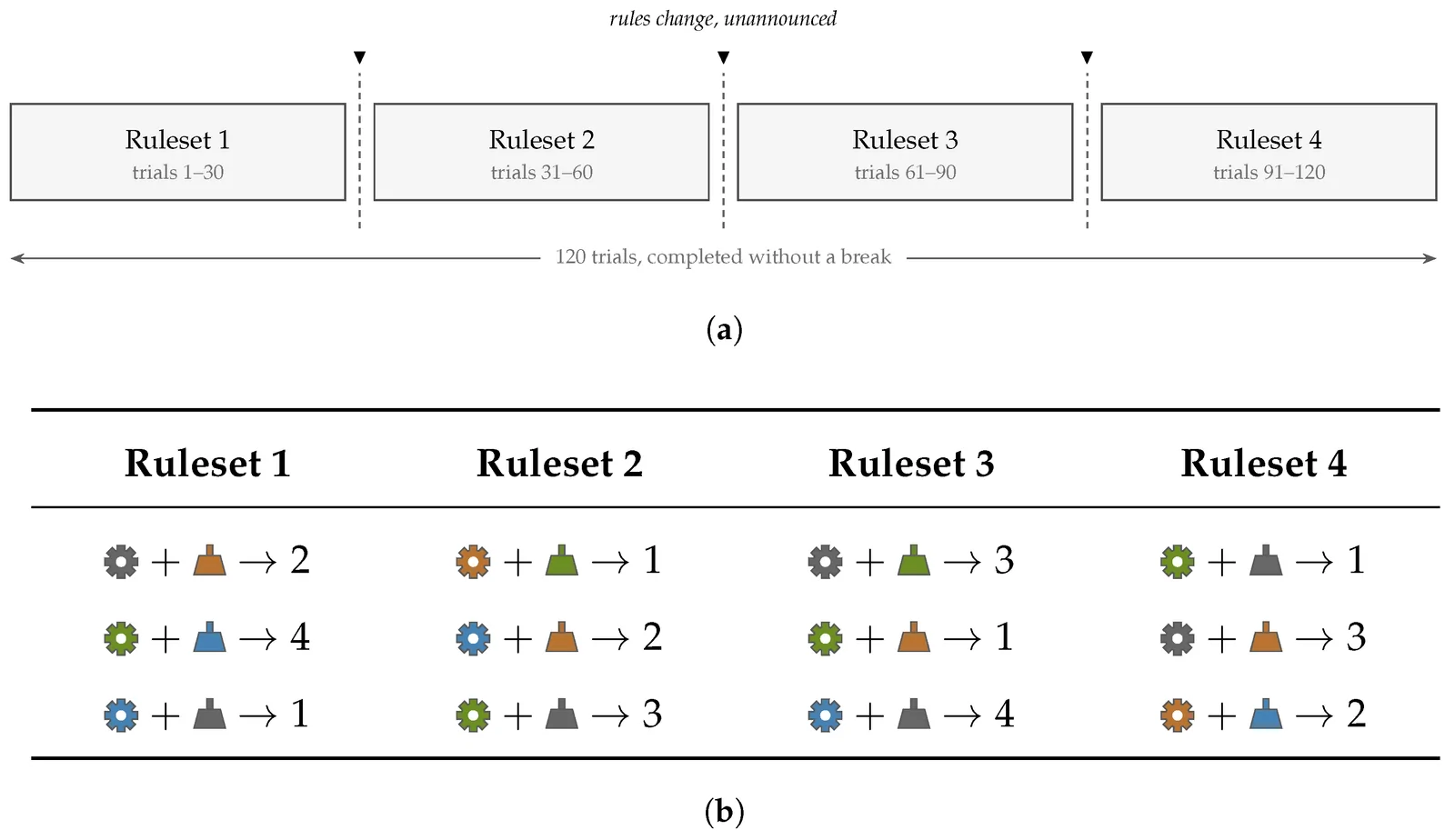
Experimental design. (**a**) The 120 trials were divided into four rulesets of 30 trials each, completed without a break; at each boundary the assembly rules changed without announcement. (**b**) The three rules in force in each ruleset, read as gear color + base color → destination bin.

The main task was a rule-based assembly and sorting paradigm consisting of four consecutive rulesets, each comprising 30 trials, for a total of 120 trials per participant without a break (Figure 2). At the start of each trial, a single colored gear appeared in a tray to the participant’s left. The participant had to pick up the gear, pick up the correct colored base as specified by the current ruleset, assemble them by bringing them together, and deposit the assembly into the correct numbered bin. A trial ended once the assembly was deposited, after which a new gear appeared immediately. The gear sequence was the same for all participants across all trials, ensuring comparable task conditions.

Each ruleset defined three rules, one per gear color active in that block. Each rule specified which base color the gear should be paired with and which bin number the assembly should be placed in. Correct and incorrect actions were distinguished by distinct auditory feedback. No other indication of correctness was given, and the task continued immediately after an incorrect sort without repeating the trial.

At the boundary between rulesets, the assembly rules changed without any prior announcement. Participants had to detect the change themselves, typically after receiving unexpected negative feedback on trials they believed were correct. This design served two purposes: to keep participants in an active learning state throughout the experiment, and to periodically induce uncertainty that would naturally elicit help-seeking behavior.

Participants could consult the current rules on the blackboard at any point during a trial. The blackboard was blank by default to prevent passive rule exposure. To make the rules appear for the first time in a trial, a participant had to look directly at the avatar’s face for at least 3 s. The rules then appeared on the blackboard and remained visible for the rest of that trial, so later consultations in the same trial required only looking at the blackboard. One exception occurred: if a base was placed in a bin without its gear, the sort was rejected and the participant remained in the same trial, but the rules disappeared and had to be activated again.At the first request after a ruleset change, the avatar additionally stated that the rules had changed.

The first episode in a trial began when the participant started looking at the avatar to activate the rules. In practice, this activation did not always consist of one uninterrupted 3 s look. Participants sometimes glanced at the still-blank blackboard to check whether the rules had appeared and then returned their gaze to the avatar. Brief tracking losses also occasionally interrupted an otherwise continuous look. Looks at the avatar separated by no more than 5 s were therefore treated as part of the same activation, with episode onset defined as the beginning of the first look. The median activation lasted 3.1 s; about 5% lasted longer than 10 s, all involving repeated look-and-check attempts.

Once the rules appeared, participants turned to the blackboard to read them. The avatar activation and this first reading were treated as a single episode when reading began within 5 s of the last look at the avatar. This occurred for 321 of the 322 activations. In the remaining case, the participant returned to the task and read the rules only 27.7 s later. The preceding avatar looks were therefore not counted as help-seeking, and the episode began with the first look at the blackboard. During reading, look-aways of up to 5 s were treated as part of the same episode, which ended with the final look at the blackboard.

Because the rules remained visible for the rest of the trial, participants could later consult them without looking at the avatar again. A return to the blackboard after more than 5 s away was counted as a new episode, beginning with the first look back.

The 5 s tolerance was derived from the observed behavior. Glances at the still-blank blackboard between avatar looks never exceeded 4.3 s, and the longest interval between the final avatar look and the first look at the displayed rules was 4.0 s. A 5 s tolerance therefore preserved these sequences as single episodes. In summary, an episode began with the behavior used to access or re-access the rules and ended when the participant stopped consulting them.

### 2.4. Data Collection

Gaze samples were logged at the application’s frame rate, which varied across recordings depending on rendering load (mean =62.51 Hz, SD=7.61 Hz). For 19 of the 21 participants, approximately half of the logged gaze samples were identical to the preceding sample, indicating that a new gaze value was available on only about half of the logged frames. Each sample contained the gaze direction vector for the left eye, right eye, and their combination in world coordinates, along with the gaze origin coordinates. Head motion was captured from the HMD pose at the same logging rate, providing continuous position and orientation data in world space. Hand motion was recorded from the Meta Quest Pro’s two 6-DoF controllers, capturing position and orientation for both the left and right hand throughout each trial.

Blood volume pulse and electrodermal activity were also recorded with an Empatica EmbracePlus wristband but, owing to incomplete coverage across participants, were not analyzed here. All features reported below derive from signals provided natively by the headset and its controllers.

### 2.5. Feature Extraction

From the three behavioral streams (eye, head, and hand), we extracted 44 features describing gaze and movement within each analysis segment (a trial or a shorter window of continuous behavior): 16 eye-tracking, 14 head-movement, and 14 hand-movement features. The number of features per modality was not specified a priori, but resulted from the signals available within each modality and the measures applicable to them. All 44 features were retained in the analyses reported here. Full definitions, including units, are provided in Appendix A.

#### 2.5.1. Eye-Tracking Features

Sixteen features were extracted from the eye-tracking stream (Table A1 in Appendix A), covering fixation behavior (four features), gaze velocity (three), gaze acceleration (three), saccade amplitude (three), gaze switching (two), and gaze transition entropy (one).

Areas of interest (AOIs) were defined within the Unity scene using collider-based object tagging, consistent with prior work using the same hardware and engine [9]. During recording, each gaze sample was annotated in real time with the AOI intersected by the participant’s gaze, drawn from the following set: the avatar, the gear, the assembly bases, the gear tray, the combined object (gear and base assembled together), the blackboard, and a residual “Environment” category for gaze directed elsewhere. The gaze-switching and entropy features described below were computed at the level of fixations, with each fixation assigned the AOI label that occurred most often among its gaze samples.

Fixations were identified using the Identification by Velocity-Threshold (I-VT) algorithm [57,58], which classifies consecutive gaze samples as part of a fixation if the angular velocity between them falls below a fixed threshold, and as a saccade otherwise. Repeated samples (Section 2.4) were first removed, retaining a sample only if its gaze direction differed by at least 0.001° from the last retained sample. Angular velocity was computed as the angular distance between the normalized gaze direction vectors of consecutive retained samples, divided by the time interval between them, and was treated as missing across intervals longer than 100 ms. A velocity threshold of 30°/s was used, consistent with the most commonly recommended value for desktop eye tracking [59] and within the 20–35°/s range identified as optimal for I-VT fixation detection in VR head-mounted displays [60]. Following the Tobii I-VT Fixation Filter [59], adjacent fixations separated by no more than 75 ms and 0.5° were merged, after which fixations shorter than 75 ms were discarded, matching the minimum duration used by Zhao et al. [60]. For each segment (trial or window), the number of fixations, their total duration, and the mean and standard deviation of fixation duration were computed.

Gaze velocity was summarized by its mean, standard deviation, and maximum value within each segment. Gaze acceleration was computed as the absolute change in velocity between consecutive velocity values, divided by the time between them, and was not computed across tracking gaps; it was likewise summarized by its mean, standard deviation, and maximum value. Saccade amplitude was computed as the angle between the mean gaze directions of consecutive fixations, and summarized by its mean, standard deviation, and maximum value. The number of changes in AOI label between consecutive fixations was counted (gaze switches), including changes to and from the Environment category, and expressed both as a raw count and as a rate per second of segment duration.

Gaze transition entropy quantifies the predictability of transitions between AOIs as a normalized Shannon entropy over a first-order Markov transition matrix, weighted by the matrix’s stationary distribution [61]. In the original formulation, self-transitions are retained, so prolonged attention to a single AOI, such as reading the rules on the blackboard, can lower entropy even though it carries little information about transitions between AOIs. We therefore used the modified gaze transition entropy (mGTE) of De Bruyne et al. [62], which removes self-transitions. For each segment, a transition matrix was built from the sequence of fixation AOI labels, self-transitions were removed, and rows were normalized to transition probabilities. The resulting entropy was normalized by the logarithm of the number of AOI states to yield a value in [0, 1], with higher values indicating a less predictable, more exploratory pattern of visual attention. Because gaze was recorded in world coordinates, it depends on both the orientation of the head and the direction of the eyes within it, so these features reflect head movement as well as eye movement. Eye-in-head eccentricity (Section 2.5.2) was computed to partly isolate the eye’s own contribution.

#### 2.5.2. Head Movement Features

Fourteen features were extracted from the head pose stream (Table A2 in Appendix A), derived from HMD position and orientation and covering translational and rotational head motion, together with the direction of gaze relative to the head. As the application’s frame rate varied across recordings (see Section 2.4), all frame-to-frame kinematic features described below were computed using the actual time interval between consecutive samples, rather than assuming a fixed sampling rate.

Head speed and acceleration were computed from frame-to-frame differences in HMD position: speed as the Euclidean distance between consecutive positions divided by the sample interval, and acceleration as the absolute frame-to-frame change in speed divided by the sample interval. Rather than being clipped, values were treated as missing when the sample interval was shorter than 5 ms (timestamp irregularities) or when they exceeded physically plausible limits (speed > 4 m/s, acceleration > 100 m/s^2^), so that tracking artifacts could not appear as a segment’s maximum. For each segment, the mean, maximum, and standard deviation of speed were computed, along with the mean and standard deviation of acceleration. The proportion of segment time during which head speed fell below 0.01 m/s was computed as the head idle fraction, weighting each frame by its duration so that the measure did not depend on the frame rate.

Head rotation speed was computed as the angular distance between consecutive HMD orientation quaternions, divided by the sample interval, and summarized by its mean, maximum, and standard deviation. Artifacts were handled as for speed, with rotation speeds above 1000°/s, well beyond the physical limits of human head movement, treated as missing. Each HMD orientation was additionally converted to yaw, pitch, and roll Euler angles in world coordinates. Yaw was unwrapped across the segment to remove ±180° wrap-around discontinuities, after which the yaw range and pitch range were computed as the difference between the maximum and minimum values observed within the segment. The number of yaw direction changes was defined as the number of sign reversals in the frame-to-frame yaw increment. Only increments of at least 1° were considered, reducing reversals caused by tracking noise. This feature captures back-and-forth head rotation, such as repeated turns between two points of interest.

Eye-in-head eccentricity was computed as the angle between the gaze direction, expressed in the head’s coordinate frame, and the head’s forward axis. It reflects how far the eyes were directed away from the center of the head’s orientation, independently of where the head itself was pointing. It was computed from the same retained gaze samples as the eye-tracking features (Section 2.5.1) and summarized by its mean and standard deviation.

#### 2.5.3. Hand Movement Features

Fourteen features were extracted from the two hand controller streams (Table A3 in Appendix A), covering the linear and rotational movement of each hand individually.

For each segment, the hand with the greater path length (excluding tracking artifacts) was labeled the active hand and the other the supporting hand, with ties resolved in favor of the right hand (none occurred). The labeling was recomputed for each trial or window and reflects which hand did more of the manipulation, not handedness, which was not assessed.

For both the active and supporting hand, speed was computed as the frame-to-frame Euclidean distance between consecutive controller positions divided by the sample interval, and acceleration as the absolute frame-to-frame change in speed divided by the sample interval. For each hand, the mean, maximum, and standard deviation of speed were computed, along with the mean and standard deviation of acceleration. Rotation speed for each hand was computed as the angular distance between consecutive controller orientation quaternions, divided by the sample interval, and summarized by its mean and standard deviation. Artifacts were handled in the same way as for the head features (Section 2.5.2).

#### 2.5.4. Performance and Context Features

Two further feature groups were derived from the task logs rather than from the behavioral streams. Unlike the features above, they depend on trial-level outcomes and on the structure of the session, and are therefore available only in the trial-level prediction task.

Performance features capture measurable aspects of what occurred during a trial: its duration, whether any incorrect sort occurred (a binary flag), the latency to the first grab action, the total number of grab actions, and three measures of blackboard consultation within that trial, namely the number of consultation episodes, their total duration, and their mean duration (Table A4 in Appendix A). Context features encode the trial’s structural position in the session, including the current ruleset block, the trial index within and across blocks, and whether the trial immediately follows a ruleset change (Table A5 in Appendix A).

### 2.6. Prediction Tasks

Prediction was evaluated at two temporal resolutions. We first considered a trial-level formulation, using completed trials to examine how non-verbal behavior compares with performance and contextual information for predicting help-seeking in the following trial. We then moved to the primary window-level formulation, which treats behavior as a continuous stream and uses only non-verbal features to predict an upcoming help-seeking episode.

#### 2.6.1. Trial-Level Setup

As an initial trial-level analysis, each completed trial constituted one observation and was used to predict whether help-seeking would occur in the immediately following trial. The non-verbal features from Section 2.5 were computed over the full duration of the trial, yielding one feature vector per trial, to which the performance and context features (Section 2.5.4) were added.

Each trial received a binary label indicating whether any help-seeking event occurred in the immediately following trial: 1 if at least one consultation took place, and 0 otherwise. Each participant’s final trial, which has no successor, was excluded, as were trials whose log-transformed duration exceeded three standard deviations from the participant’s own mean (51 trials removed). The resulting dataset comprised 2448 trials across 21 participants, with 293 positive labels (12.0%).

#### 2.6.2. Window-Level Setup

**Figure 3:**
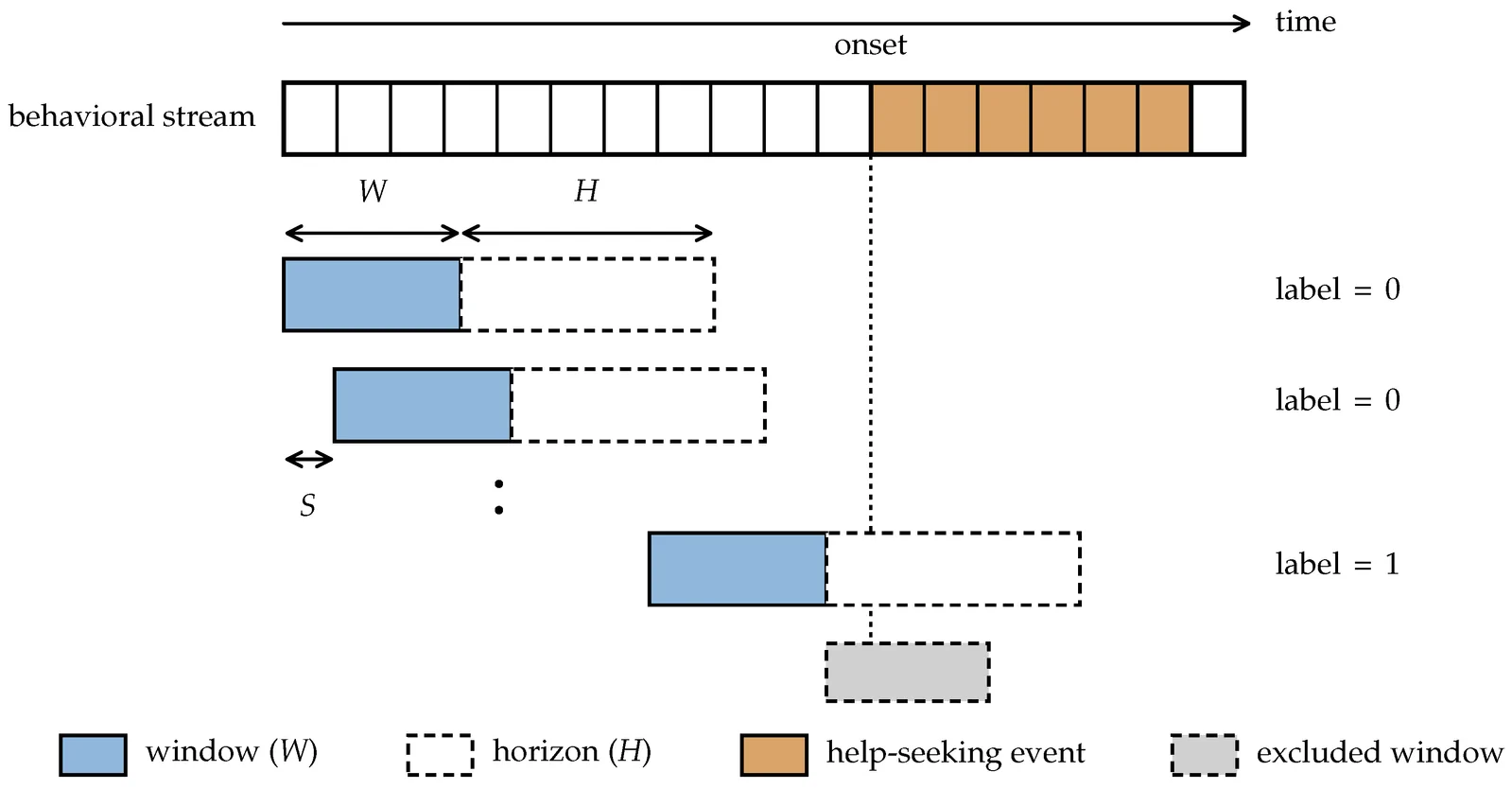
Construction and labeling of window-level observations. A window of length *W* slides across the behavioral stream at stride *S*, and is labeled 1 if a help-seeking event begins within the following horizon of *H* seconds, and 0 otherwise. Windows overlapping an ongoing event were excluded. Cell widths are schematic and not to scale.

In the window-level setup, a fixed-length behavioral window is slid continuously across each participant’s session at a fixed stride, with each window constituting one observation (Figure 3). Unlike the trial-level setup, windows are not constrained to trial boundaries and may span two consecutive trials, preserving the continuity of the behavioral signal.

Any window overlapping a help-seeking episode was excluded, ensuring that the model was exposed only to behavior preceding an episode rather than to the help-seeking action itself. Episode boundaries followed the definition in Section 2.3: the first episode in a trial began with the avatar-directed activation, whereas later episodes began with a return to the already visible blackboard.

Each remaining window received a binary label based on the fixed lookahead period following its end, referred to as the prediction horizon. A window was labeled positive (1) if a new help-seeking episode began within this horizon and negative (0) otherwise. The model therefore predicts whether a new episode will begin shortly after the observed behavioral window rather than detecting an episode already in progress.

The non-verbal features described in Section 2.5 were extracted from the data within each window, producing one feature vector per window. No performance or context features were included. Performance features depend on completed-trial outcomes, while context features encode the trial’s position in the experimental session rather than continuously observed behavior.

The window length, stride, and horizon are design parameters of the prediction task; their effect on prediction performance was evaluated empirically, and results are reported in Section 3.

### 2.7. Model Evaluation

All models were evaluated using leave-one-subject-out (LOSO) cross-validation. In each fold, one participant was held out for testing while the remaining participants were used for training. This procedure was repeated until every participant had served as the test participant exactly once. LOSO is commonly used when the goal is subject-independent prediction because it evaluates performance on previously unseen individuals rather than on additional samples from participants observed during training [63].

Because help-seeking was relatively rare, overall accuracy was not informative. We therefore report balanced accuracy (BA), for which chance is 0.5 regardless of class imbalance, together with the $F_{1}$ score.

For the window-level configuration sweep, the proportion of positive windows varied across window lengths, horizons, and strides. We therefore used AUC-PR as the primary threshold-independent metric for imbalanced classification [64] and report it together with its chance level, which equals the positive-class prevalence [65]. Because this baseline differs between configurations, we additionally report lift (AUC-PR divided by its chance level) and AUC-ROC, which is independent of class prevalence. AUC-ROC was used when directly comparing conditions with different positive rates.

Once a decision threshold was selected for the final models, we additionally report balanced accuracy, $F_{1}$, recall, and precision.

To complement the window-level metrics with event-level measures, we evaluated the thresholded held-out predictions of the final nested LOSO model at the level of help-seeking episodes. A help-seeking episode was considered detectable if at least one eligible analysis window ended within the 3 s prediction horizon before its onset, and anticipated if at least one positive prediction occurred in that interval. For anticipated episodes, we report the earliest qualifying prediction relative to onset. To quantify false alerts, consecutive positive predictions were merged into alert episodes; an alert was counted as matched when a help-seeking episode occurred within the 3 s prediction horizon, and event-level precision was the proportion of alert episodes that were matched. As a temporal baseline that accounts for dependence between neighboring windows, each participant’s predictions were circularly shifted in time over 1000 iterations. This preserved the number and temporal structure of alerts within each session while breaking their alignment with the actual help-seeking episodes; the event detection rate and event-level precision were recomputed after each shift.

## 3. Results

We first characterize how help-seeking behavior varied across participants, then evaluate trial-level and window-level prediction of future help requests. We then examine how far in advance of a request the predictive signal is available, and describe the behavioral changes that precede help-seeking.

### 3.1. Heterogeneity in Help-Seeking Behavior

Across the 21 participants (120 trials each; 2520 trials in total), 321 trials contained at least one help-seeking episode. Each of these trials contained one initial consultation, and because rule checks separated by no more than 5 s were merged into one episode (Section 2.3), most of them (287 of 321) contained only this episode. The remaining 34 trials also contained one or more later returns to the rules (54 returns in total), yielding 375 help-seeking episodes overall. These trials contained 322 rule activations, because in one trial the rules were activated a second time after such a rejected sort (Section 2.3). The number of episodes per participant ranged from 6 to 54 (median 16). Episodes within a trial, and trials within a participant, are not independent observations. For statistical comparisons, the participant was therefore used as the unit of analysis, while predictive performance was evaluated using participant-held-out data throughout.

The proportion of trials on which a participant sought help at least once averaged 12.7±9.0% but ranged from 4.2% to 44.2%, a 10.6-fold difference between the most and least frequent help-seekers (coefficient of variation = 0.71). The median rate was 10.0% (IQR 8.3–13.3%), and the distribution was right-skewed: most participants fell between roughly 4% and 24%, with one pronounced outlier (P18) consulting the blackboard on 44% of trials.

### 3.2. Trial-Level Prediction of Help-Seeking

We first asked whether a learner’s help-seeking on the *next* trial can be predicted from features of the *current* trial. Labels were assigned from the complete trial sequence; each participant’s final trial, which has no successor, was then excluded, as were trials with outlying durations (Section 2.6). This left 2448 trials, of which 293 (12.0%) were followed by a help request. To avoid tying the result to one model’s inductive bias, we compared a logistic-regression baseline (linear and directly interpretable) with two tree-ensemble methods that perform well on tabular data [66,67]: random forest (bagged trees) and XGBoost (gradient-boosted trees). All models used class-balanced weighting and fixed hyperparameters without tuning, and predictions were thresholded at a fixed probability of 0.5, so that no decision threshold was tuned on held-out data. On the full non-verbal feature set, logistic regression performed best (BA = 0.597±0.105, $F_{1}\;=\;0.253\pm 0.158$). At this threshold the two tree ensembles rarely predicted a help-seeking trial (random forest: BA = 0.514, $F_{1}\;=\;0.083$; XGBoost: BA = 0.526, $F_{1}\;=\;0.118$), because their predicted probabilities for the minority class seldom exceeded 0.5. Logistic regression was therefore used for the remaining trial-level analyses.

**Figure 4:**
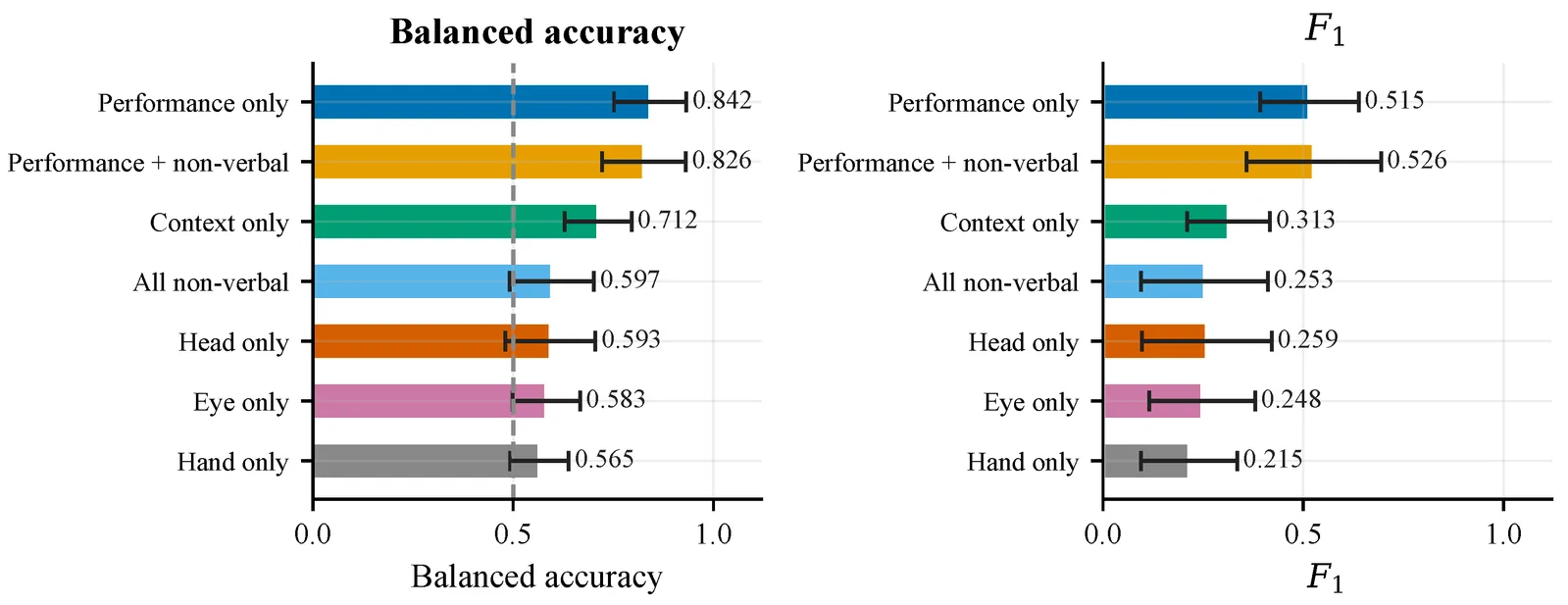
Trial-level prediction by feature group (logistic regression, LOSO, fixed decision threshold of 0.5). Balanced accuracy (**left**) and $F_{1}$ (**right**); error bars are SD over 21 folds, and the dashed line marks chance-level balanced accuracy (0.5). Performance and context features dominate, while the non-verbal group reaches BA = 0.597.

Predictive performance was dominated by the performance feature group (Figure 4). Performance features alone achieved the highest scores among the individual feature groups (BA = 0.842±0.090, $F_{1}\;=\;0.515\pm 0.123$), followed by the context features (BA = 0.712±0.084, $F_{1}\;=\;0.313\pm 0.103$). Both outperformed the full non-verbal feature set (BA = 0.597±0.105, $F_{1}\;=\;0.253\pm 0.158$) as well as each individual non-verbal modality (BA 0.565–0.593). Combining the seven performance features with the 44 non-verbal features (51 features in total) yielded BA = 0.826±0.105 and $F_{1}\;=\;0.526\pm 0.168$.

Adding the non-verbal features did not significantly change balanced accuracy (higher in 13 of 21 folds, lower in 6, tied in 2; two-sided Wilcoxon signed-rank test, W=93, p=0.94, rank-biserial r=0.02) but produced a small, consistent increase in $F_{1}$ (higher in 15 folds, lower in 4, tied in 2; W=41, p=0.030, r=0.57). Because balanced accuracy and $F_{1}$ emphasize different aspects of classifier performance, improvements in one metric do not necessarily translate into improvements in the other [68]. At the trial level, the non-verbal features therefore added at most a modest amount of predictive information beyond the performance features.

**Figure 5:**
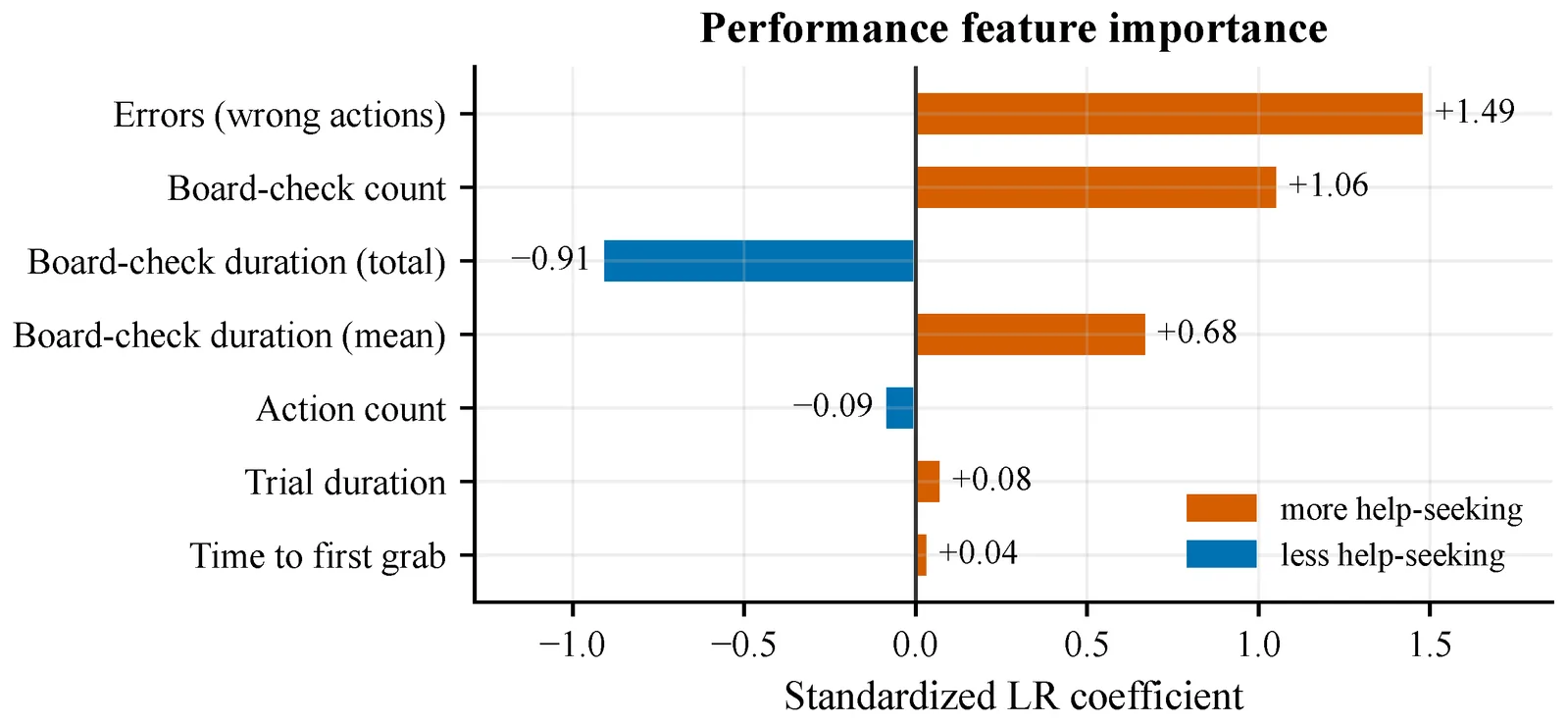
Standardized logistic-regression coefficients for the seven performance features (fitted to all 2448 trials). Positive values indicate a higher likelihood of help-seeking on the next trial. Errors are the strongest predictor; the three board-check features are collinear and should be read jointly.

The two leading groups, however, are less suitable for real-time anticipation. Context features (block index, trial number, and a new-block indicator) reflect the structure of the experimental design rather than the learner’s ongoing behavior, while performance features summarize events that have already occurred within the completed trial. Within the performance group, the strongest predictor was whether the learner made an error (standardized β=+1.49), followed by the number of board checks (β=+1.06) and their total duration, which acted in the opposite direction (β=−0.91); the three board-check measures are collinear and should be read jointly (Figure 5). Error is known only once a trial has ended, and the board-check measures count consultations that are themselves help requests, so both describe the help-seeking process rather than the behavior preceding it. A trial-level summary also averages behavior over the whole trial, so any short-lived change shortly before a help request is lost. We therefore reframe the task at the window level (Section 2.6.2): instead of one prediction per trial, we predict from short overlapping windows of behavior whether a help request will begin within the next few seconds.

### 3.3. Window-Level Prediction of Help-Seeking

We next evaluated whether upcoming help-seeking could be predicted continuously from non-verbal behavior using the window-level setup described in Section 2.6.2. We varied the window length (*W*, 3–7.5 s), the prediction horizon (*H*, 3–6 s) and the stride (*S*, 1–2 s), and compared four classifiers (logistic regression, random forest, XGBoost and LightGBM), each with fixed hyperparameters and class-balanced weighting. This gave 480 configurations, each evaluated with LOSO cross-validation.

Positive windows made up between 5.2% and 11.3% of observations, depending on the configuration. Since the chance level of AUC-PR equals this positive rate, raw AUC-PR values cannot be compared directly across configurations. For each configuration we therefore computed its own chance level and report AUC-PR together with its lift over chance (mean AUC-PR across held-out participants divided by their mean chance level). We also report AUC-ROC, which does not depend on the positive rate (Section 2.7).

**Figure 6:**
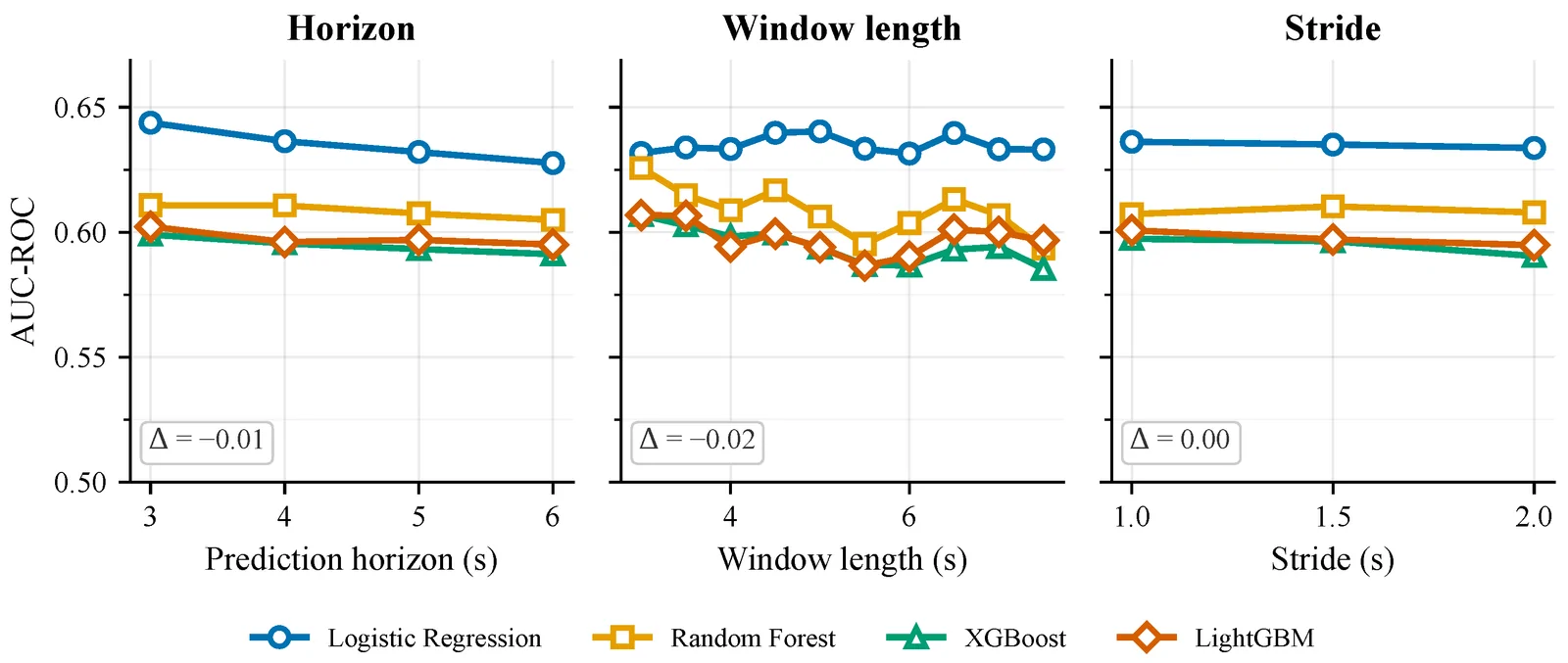
Effect of prediction horizon, window length and stride on AUC-ROC (LOSO, each point is the mean over the other two parameters). AUC-ROC is independent of the positive rate and therefore comparable across horizons. The axis starts at chance level (0.5). Δ is the change from the smallest to the largest parameter value, averaged over the four classifiers.

The prediction horizon affected the two metrics differently (Figure 6). A longer horizon means that more windows are followed by a request within it, so the chance level rose from 0.060 at 3 s to 0.111 at 6 s. Raw AUC-PR rose with it, from 0.115 to 0.179 on average, but AUC-ROC stayed almost the same (0.614 and 0.605), and lift fell from 1.93 to 1.62. The higher AUC-PR at longer horizons therefore reflected a higher chance level rather than better ranking of windows. Window length had a small effect, with shorter windows performing slightly better (AUC-ROC 0.618 at 3 s and 0.602 at 7.5 s, lift 1.85 and 1.71). Stride had almost no effect on AUC-ROC (0.610 to 0.607 across 1–2 s).

We then identified the configurations with the highest lift and the highest AUC-ROC across the full sweep (Table 1). For every classifier, both optima occurred at the 3 s horizon, which targets an imminent request while leaving time for a system to respond. Overall, the highest lift was obtained with logistic regression at W=3 s and S=2 s (AUC-PR = 0.160 against a chance level of 0.064, lift 2.50, AUC-ROC 0.647), and the highest AUC-ROC with logistic regression at W=4.5 s and S=2 s (AUC-PR =0.148 against 0.061, lift 2.42, AUC-ROC 0.660). Both configurations were carried forward. Because they were selected from the sweep, the values above should be read as an upper estimate. Across all 480 configurations, AUC-ROC ranged from 0.554 to 0.660 (median 0.605) and lift from 1.40 to 2.50 (median 1.73). Both configurations were then evaluated with nested cross-validation, in which hyperparameters and the decision threshold were selected on training participants only (Section 3.4).

### 3.4. Hyperparameter Tuning and Threshold Selection

The two configurations identified in the sweep were evaluated with nested LOSO cross-validation. In each outer fold, one participant was held out, and LOSO over the remaining 20 participants was used to select the hyperparameters and the decision threshold. The model was then retrained on all 20 and evaluated once on the held-out participant, so that the test participant never influenced any choice. For logistic regression, we searched the regularization strength (C∈{0.0001, 0.001, 0.01, 0.1}) and the penalty (L1 or L2), selecting the combination with the highest mean AUC-PR across the inner folds. Unlike in the sweep, all candidate combinations were evaluated on the same windows and therefore shared the same chance level, so AUC-PR could be compared directly. All 44 features were standardized within each training fold, and classes were weighted inversely to their frequency. The decision threshold was set to the value that maximized $F_{1}$ on the pooled out-of-fold predictions for the 20 training participants, balancing recall against precision without assigning a cost to each type of error, and was applied unchanged to the held-out participant.

The selected settings were stable across folds. For the 3 s window, the L2 penalty was chosen in all 21 folds, with C=0.001 in 15 folds, C=0.01 in 5 and C=0.1 in 1, and the threshold ranged from 0.54 to 0.64 (median 0.58). For the 4.5 s window, C=0.01 with the L2 penalty was chosen most often (12 folds), and the threshold ranged from 0.51 to 0.67 (median 0.59).

Table 2 summarizes performance on the held-out participants. The two configurations did not differ significantly on any metric (paired Wilcoxon signed-rank tests across participants, all p>0.08). We therefore report the 3 s window as the primary model, as it requires less behavioral history before a prediction can be made. With this configuration, the model reached an AUC-PR of 0.159±0.063 against a chance level of 0.064, a lift of 2.5, and an AUC-ROC of 0.667±0.086. At the selected threshold, it detected 40% of upcoming requests (recall 0.399±0.189). Of its alerts, 13% were followed by a request (precision 0.127±0.070), about twice the rate expected by chance (6.4%), giving a balanced accuracy of 0.591±0.069 and an $F_{1}$ of 0.179±0.078. Lowering the threshold would raise recall at the cost of precision.

At the event level, 35 of the 375 help-seeking episodes could not be anticipated because no eligible analysis window ended within the 3 s before onset; these episodes followed shortly after a previous episode, so all preceding windows had been excluded. Among the remaining 340 detectable episodes, the model anticipated 157 (46.2%). For these episodes, the earliest qualifying prediction occurred a median of 1.61 s before onset (IQR 0.91–2.23 s; mean 1.55±0.85 s).

Across all held-out predictions, the model produced 824 alert episodes. Of these, 157 were matched to a help-seeking episode within the 3 s prediction horizon, while the remaining 667 were false alerts, giving an event-level precision of 0.19. When each participant’s predictions were circularly shifted in time (1000 iterations), 29% of detectable episodes were anticipated and event-level precision was 0.12. None of the shifted iterations reached the observed detection rate or precision (p=0.001 for both).

**Figure 7:**
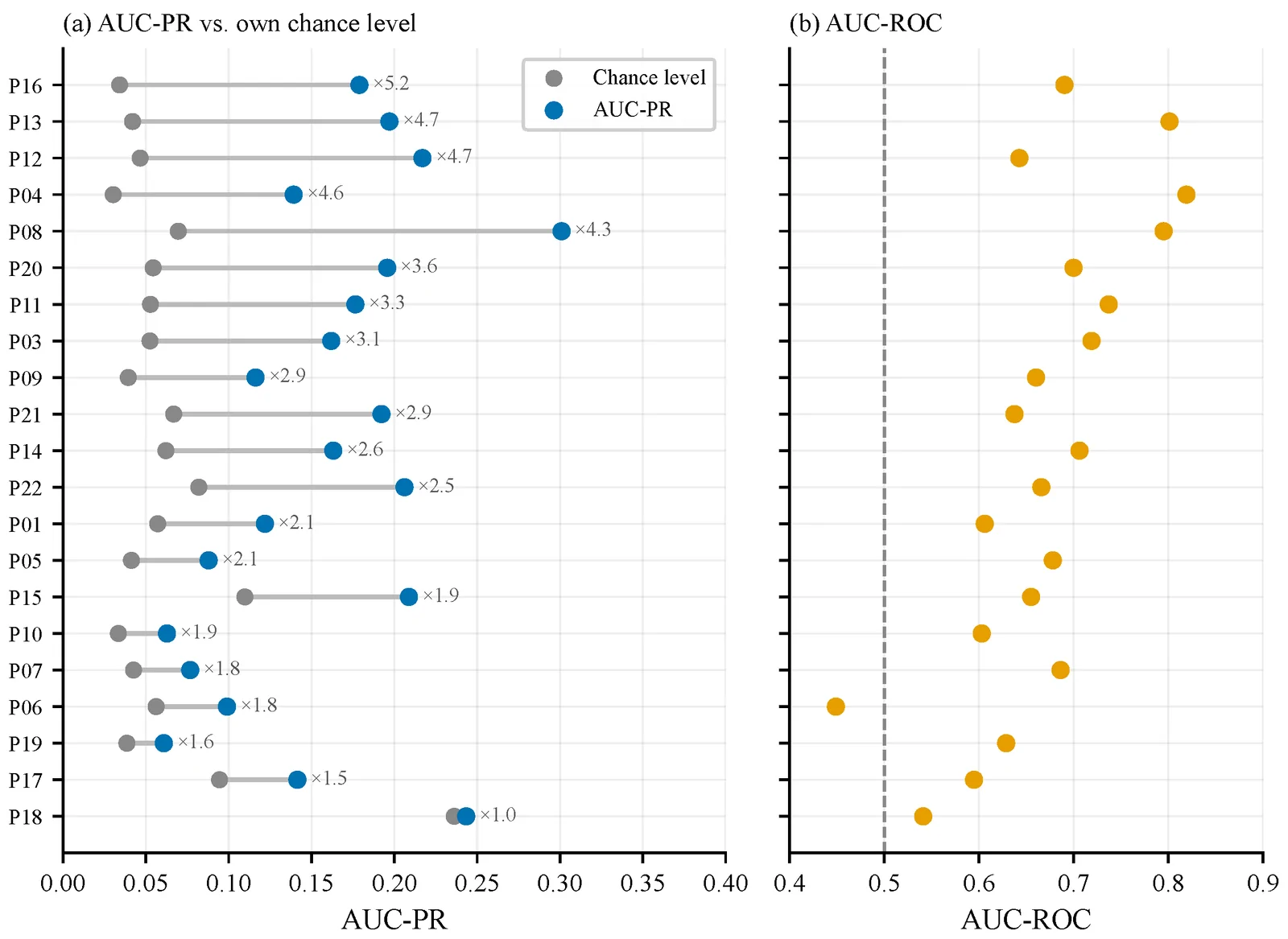
Performance for each held-out participant under nested LOSO (W=3 s, H=3 s, S=2 s), sorted by lift. (**a**) AUC-PR (blue) against the participant’s own chance level (grey), with lift on the right. (**b**) AUC-ROC; the dashed line marks chance (0.5).

Performance on each held-out participant is shown in Figure 7. The gain over chance varied considerably between participants, with lift ranging from 1.0 to 5.2 and AUC-ROC from 0.45 to 0.82. This variation was consistent across the two configurations (Spearman ρ=0.79 for per-participant AUC-ROC), indicating that it reflects differences between participants rather than noise in a single analysis. Part of it relates to how often participants sought help. Their chance level ranged from 0.03 to 0.24, and the participant who sought help most often (P18) showed almost no gain over chance (lift 1.03). The same general model therefore predicted help-seeking well for some learners and only marginally for others.

### 3.5. Feature Importance

Having fixed the operating model, we asked which behavioral features it relies on. We used a single fixed model, the combination selected most often in the nested procedure (C=0.001, L2 penalty, 15 of 21 folds), trained on the same windows (W=3 s, H=3 s, S=2 s) under the same LOSO protocol. Importance was measured by permutation. The values of a feature in the held-out participant were shuffled, breaking their relation to the labels, and the resulting drop in AUC-ROC was averaged over 20 shuffles. AUC-ROC was used rather than AUC-PR because it does not depend on the proportion of positive windows, which differed considerably between participants, so drops could be compared and averaged across them. Drops were tested against zero with a one-sided Wilcoxon signed-rank test across the 21 participants, using one value per participant, and *p*-values were corrected with the Benjamini–Hochberg procedure, with the adjusted values reported as *q*-values.

**Figure 8:**
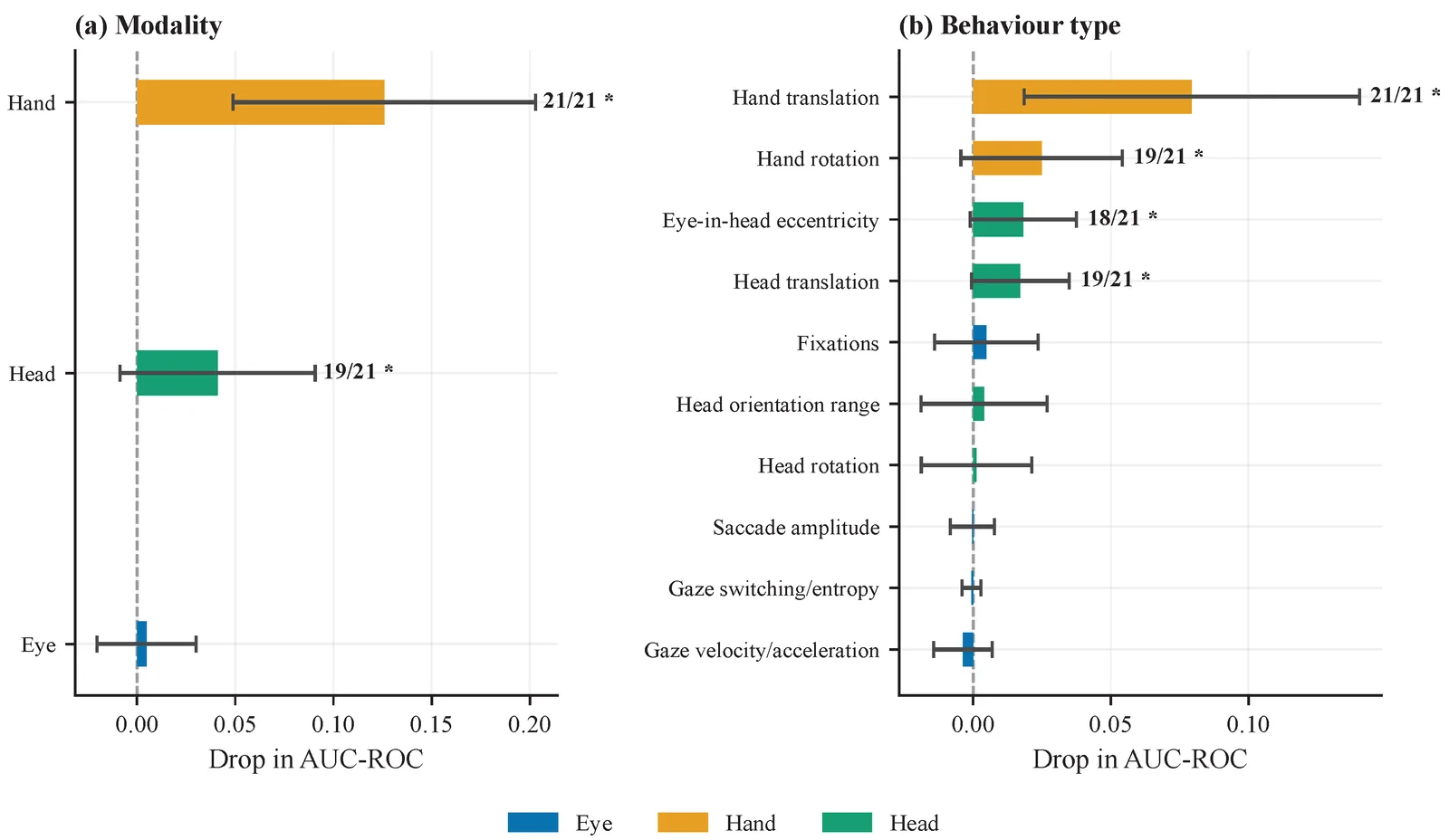
Grouped permutation importance. Drop in AUC-ROC when all features of a group were shuffled jointly in the held-out participant, grouped by (**a**) modality and (**b**) behavior type. Bars show the mean and error bars the SD across 21 participants. Numbers give the participants whose AUC-ROC dropped, and asterisks mark a significant drop (one-sided Wilcoxon signed-rank test, Benjamini–Hochberg corrected, q<0.05).

Many features were strongly correlated (21 of the 946 feature pairs had |ρ|≥0.85). When one feature of such a pair is shuffled, the model still receives similar information from the other, so its individual importance is underestimated. We therefore also permuted groups of features jointly. Groups were defined by what the features measure rather than by their correlations, at two levels. By modality, features formed an eye (16), a head (14) and a hand group (14). By behavior type, they formed ten groups (Figure 8b). These groups also captured the correlation structure, since 20 of the 21 highly correlated pairs fell within the same behavior-type group.

Hand features contributed most to prediction, followed by head features, whereas permuting the eye feature group did not produce a significant reduction in AUC-ROC (Figure 8a). Permuting the hand features reduced AUC-ROC in all 21 participants (0.126±0.077), and permuting the head features in 19 of 21 (0.041±0.050). Among behavior types, four groups contributed significantly, namely hand translation, hand rotation, eye-in-head eccentricity and head translation (Figure 8b). For each of these four groups, shuffling all members together reduced AUC-ROC 1.2 to 2.3 times more than the sum of the drops obtained by shuffling each member alone, consistent with correlated features partly substituting for each other.

Individually, six of the 44 features had a significant effect, all with small drops (at most 0.014). The largest was mean eye-in-head eccentricity, followed by the mean and standard deviation of active-hand speed, the mean rotation speed of both hands, and the standard deviation of supporting-hand rotation speed.

### 3.6. Time Course of Behavior Before a Help Request

The permutation analysis identified the behaviors the model relied on most, but not how these behaviors unfolded before a help request. We therefore examined how the most important signals developed over the 5 s preceding each request, and compared them with the same signals at ordinary moments of the task. This shows how behavior before a request differs from ordinary behavior, and when these differences emerge.

We focused on the signals that contributed most to prediction (Section 3.5): the speed of the active hand, representing the most important modality, and eye-in-head eccentricity. To place these changes in time relative to the request itself, we also included head yaw range. A help request began with a turn of the head toward the avatar or the blackboard, so head yaw range marks when the request starts in behavior, independently of the logged onset. For each of the 375 help-seeking episodes, each signal was aligned to the episode onset and computed at 0.1 s steps from 5 s before onset to onset, each value summarizing the preceding 0.5 s: the mean speed of the active hand (the hand that moved more in these 5 s), the mean eye-in-head eccentricity, and the range of head yaw. As a reference, the same curves were computed for randomly chosen moments without help-seeking. For each participant, we drew five random moments per help request from that participant’s session, accepting only moments at least 5 s away from any help-seeking episode, and treated each as if it were a request onset. Curves were averaged within each participant, separately for help requests and random moments, and then across the 21 participants, so that participants with many requests did not dominate.

The start of the turn was defined from the head yaw curve as the first time point from which yaw range remained more than 3 SD above its baseline level (mean over −5 to −2 s) until the request. Two within-participant comparisons were made for each signal, both with a Wilcoxon signed-rank test. First, to test whether behavior before a request differed from ordinary behavior, we compared the mean before the turn with the same period for the random moments. Second, to test whether it changed over the seconds before a request, we compared the mean over −5 to −3 s with the mean over the period between −1.5 s and the start of the turn.

**Figure 9:**
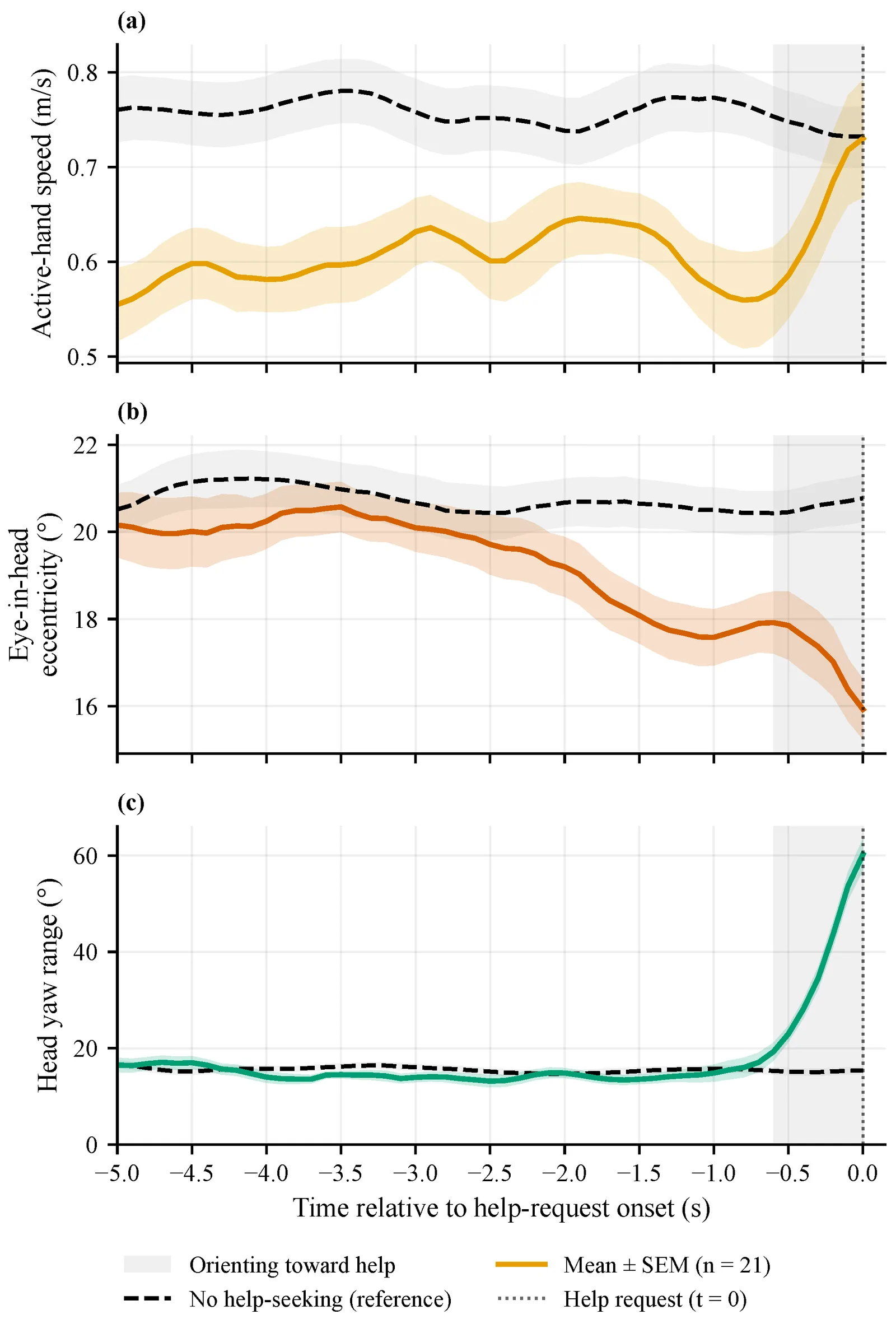
Behavior in the 5 s before a help request (solid) and before random moments without help-seeking (dashed): (**a**) active-hand speed, (**b**) eye-in-head eccentricity, (**c**) head yaw range. Mean ± SEM across participants (n=21); the grey area marks the turn toward the source of help.

Head yaw range followed the reference closely at about 15° until 0.6 s before the request and then rose steeply, reaching about 60° at onset (Figure 9c). At the group level, the increase in head yaw was therefore confined to approximately the final 0.6 s before onset. Before it, head yaw range did not differ from the reference (14.7° vs. 15.6°, p=0.15) and did not change over time (15.1° and 14.9°, p=0.61).

The active hand, in contrast, moved more slowly before a request than at random moments throughout the 5 s (Figure 9a; 0.60 vs. 0.76 m/s, lower in 19 of 21 participants, p<0.001). This difference was already present 5 s before the request and did not grow over time (0.59 and 0.59 m/s, p=0.86). During the turn, hand speed increased and reached the level of the reference at onset.

Eye-in-head eccentricity was close to the reference until about 3.5 s before the request and then decreased, while the reference remained at about 20.5° (Figure 9b). Before the turn, it was lower than the reference (19.5° vs. 20.8°, lower in 17 of 21 participants, p<0.001), and it was lower in the period just before the turn than 3–5 s earlier (17.8° vs. 20.2°) in 20 of the 21 participants (p<0.001), before decreasing further during the turn itself.

Behavior before a help request therefore differed from ordinary behavior before the turn toward the source of help. The active hand moved more slowly throughout the 5 s before a request, and from about 3.5 s eye-in-head eccentricity decreased progressively, whereas head yaw range differed from the reference only during the final 0.6 s. The two signals that contributed most to prediction thus differed from ordinary behavior before the turn, whereas head yaw range showed no significant individual permutation importance and differed from ordinary behavior only during the turn. In the next section, we test whether prediction depends on the turn by excluding windows that overlap it.

### 3.7. Prediction Before the Orienting Movement

To test whether prediction depended on the turn toward the source of help, we repeated the analysis after excluding all windows whose end fell within a guard band *g* before the next request onset, from both training and test data. The guard bands followed from the time-course analysis (Section 3.6). The group-level trajectory indicated that the orienting movement emerged approximately 0.6 s before onset, so g=0.6 s excluded windows ending within this final orienting interval. A guard band of g=1 s added a more conservative margin for the 0.5 s smoothing window and for variation between episodes. Because excluding windows also lowers the proportion of positive windows, we report AUC-ROC, which does not depend on this proportion, and compared each guard band with a control in which the same number of positive windows was removed at random within each participant (20 repetitions). The model and its hyperparameters were those used for feature importance (C=0.001, L2 penalty), and the decision threshold was chosen within each fold from the training participants only, as in the nested analysis.

Excluding windows from the final orienting interval reduced performance only modestly (Table 3). AUC-ROC decreased from 0.673 to 0.650 at g=0.6 s, somewhat more than random removal of the same number of windows (0.671; paired Wilcoxon p=0.04), indicating that windows ending during the turn were easier to recognize. Extending the guard band to 1 s did not reduce performance further (0.649), and the difference from random removal was no longer significant (p=0.10). AUC-ROC remained above chance for 21 and 20 of the 21 participants at the two guard bands, respectively. At the selected threshold, the model detected 25–30% of upcoming requests after excluding the final orienting interval, compared with 44% without a guard band, while the precision of its alerts remained about 1.7 times the chance level, compared with 2.0 times. The turn toward the source of help therefore contributed to the model’s practical performance, but a substantial proportion of the model’s ability to distinguish upcoming requests remained in behavior preceding the turn.

### 3.8. Behavioral Signature

Independently of the predictive model, we examined whether behavioral features differed between windows followed by a help-seeking episode within the prediction horizon and those that were not (W=3 s, H=3 s, S=2 s). Because multiple, temporally overlapping windows were extracted from each participant, windows cannot be treated as independent observations. We therefore aggregated to the participant level: for each participant and feature, we computed the median across their positive windows and the median across their negative windows, yielding 21 paired observations per feature, which were compared with a two-sided Wilcoxon signed-rank test. Since all 44 features were tested simultaneously, *p*-values were adjusted using the Benjamini–Hochberg procedure, with the adjusted values reported as *q*-values. Effect sizes are reported as matched-pairs rank-biserial correlations *r*, together with the median within-participant difference Δ as a measure of magnitude. We also report $n_{\mathrm{dir}}$, the number of participants whose within-participant difference was in the same direction as the reported effect. This comparison is descriptive: it characterizes how behavior before a request differed from behavior at other times, without controlling for where in the task these windows occurred.

Of the 44 features, 30 differed significantly (q<0.05; Table 4), with |r|≥0.53 for all significant features, and they formed a single consistent pattern: behavior slowed down before a request. All 14 hand features differed, all in the direction of less movement. The active hand moved more slowly (median Δ=−0.19 m/s, r=−0.95) and rotated more slowly (Δ=−33 °/s, r=−0.96), and the same held for the supporting hand, in 16 to 19 of the 21 participants. Ten of the 14 head features differed. The head moved and rotated more slowly (mean speed Δ=−0.05 m/s, r=−0.94; mean rotation speed Δ=−5.3 °/s, r=−0.87), the range of vertical head rotation narrowed ($\Delta \;=\;-5.1^{{}^{\circ}}$, r=−0.80), and mean eye-in-head eccentricity decreased ($\Delta \;=\;-1.0^{{}^{\circ}}$, r=−0.80), indicating that gaze stayed closer to the head’s forward direction. The range of horizontal head rotation did not differ (q=0.12). Six of the 16 eye features differed, indicating more stable gaze: total and mean fixation duration increased (total Δ=+134 ms per 3 s window, r=+0.78), and gaze velocity decreased (r=−0.53 to −0.65), whereas gaze switching, transition entropy and saccade amplitude did not differ.

The features that differed most consistently here, hand movement, head translation and eye-in-head eccentricity, were also those the predictive model relied on (Section 3.5), whereas horizontal head rotation, which reflects the turn toward the source of help, did not differ.

## 4. Discussion

Training in immersive virtual reality (VR) offers an opportunity to continuously observe learner behavior without disrupting the task. Yet support still often depends on learners initiating contact with a tutor, even though help is not always sought when needed. Using performance data to adapt support is increasingly common [69,70,71], whereas the potential of non-verbal cues, such as how learners move and where they look, to indicate an emerging need for help remains less explored. We therefore examined whether eye, head, and hand behavior recorded during a VR assembly task could anticipate an upcoming help request before the request itself began.

At the trial level, performance features predicted help-seeking best (balanced accuracy 0.84), followed by context features (0.71), whereas non-verbal features reached 0.60. Adding non-verbal features to performance features did not improve balanced accuracy (0.83, p=0.94) and produced only a small increase in $F_{1}$ (0.515 to 0.526, p=0.030). However, context features partly reflect the fixed structure of the experiment, while performance features such as errors and previous rule consultations become available only after relevant events have occurred. A trial-level prediction also indicates only that help will occur somewhere in the following trial, not when. We therefore reframed the prediction problem at the window level, predicting from short segments of ongoing behavior whether a request would follow within the next few seconds.

At the window level, non-verbal behavior carried a modest but consistent predictive signal. Under nested cross-validation, the final model, using a 3 s window, a 3 s prediction horizon and a 2 s stride, reached an AUC-PR of 0.159 against a chance level of 0.064, or about 2.5 times chance, and an AUC-ROC of 0.667. At the selected threshold it detected 40% of positive windows (recall 0.399), and 13% of positive predictions were followed by a request (precision 0.127). At the event level, the model anticipated 46% of requests for which a prediction was possible, with the earliest qualifying prediction occurring a median of 1.61 s before onset; event-level precision was 0.19. This answers our first research question: non-verbal behavior alone carries information about upcoming help-seeking above the chance level, but the signal remains weak. With many requests missed and many alerts false, the model is not yet sufficient on its own to guide intervention.

For comparison, gaze-based prediction of the onset of item selection in VR reached about 2.7 times its chance level in AUC-PR and an AUC-ROC of 0.77 [45], with models trained and tested within each participant and a prediction horizon of 200 ms. Detectors of confusion and frustration evaluated on unseen learners reached AUC-ROC values of 0.65 and 0.63 [72], and gaze-based detection of mind wandering in new users reached AUC-ROC values between 0.57 and 0.72 [73]. These results provide useful context rather than a direct benchmark, since the studies differ in the behavior being predicted, how far ahead prediction is made, and whether models are evaluated within or across participants.

The time-course analysis helped distinguish behavioral changes preceding help-seeking from the final movement used to initiate the request. Active-hand speed was lower than at ordinary moments throughout the 5 s before a request. Eye-in-head eccentricity began to decrease about 3.5 s before onset, indicating that gaze became progressively more aligned with the head’s forward direction. In contrast, head yaw range closely followed ordinary behavior until approximately 0.6 s before the request, when it rose sharply as participants turned toward the avatar or blackboard. The two signals with the greatest individual permutation importance therefore differed from ordinary behavior before this orienting movement, whereas horizontal head rotation itself contributed little to prediction.

The guard-band analysis confirmed that prediction was not explained solely by this final turn. Excluding windows from the final orienting interval identified by the group-level time course reduced AUC-ROC from 0.673 to 0.650. Extending the guard band to 1 s produced almost no further reduction (0.649). The effect on thresholded detection was larger: recall fell from 44% to 25–30%, while precision remained approximately 1.7 times its chance level. The orienting movement therefore provides useful information for detecting an imminent request, but much of the model’s ability to distinguish upcoming requests remains in behavior occurring before the turn.

The model-independent analysis showed that these earlier changes were part of a broader behavioral pattern. Thirty of the 44 features differed significantly between windows with and without upcoming help-seeking, with |r|≥0.53 for all significant features. Both hands moved and rotated more slowly, and head translation and rotation also decreased. Gaze became more stable, with longer fixations, lower gaze velocity, and lower eye-in-head eccentricity. Horizontal head range, gaze switching, transition entropy, and saccade amplitude did not differ. Most participants showed the same direction of change, indicating that the pattern was not driven by a small number of individuals. Together with the time-course analysis, these results describe a period of reduced movement and more head-centered gaze before the final turn toward help.

This two-part structure is consistent with Nelson-Le Gall’s staged model of help-seeking [35]. The period of reduced movement and head-centered gaze is consistent with the earlier stages, in which learners become aware of a need for help through metacognitive monitoring and decide whether to act on it, although our data cannot separate these two stages from each other. The orienting turn is more consistent with the later stages, in which a source of help is identified and the request is initiated. The behavioral changes preceding the turn therefore suggest that the visible help-seeking act is the endpoint of a longer process rather than its beginning.

The observed slowing should not, however, be interpreted as a direct measure of any particular cognitive state. Movement can become less complex under increased cognitive demand [74], and hand-motion measures have been used as indicators of cognitive load in assembly tasks similar to ours [75]. The gaze pattern points in a similar direction. Fixations lengthened, as reported under higher cognitive load [76], and gaze stayed closer to the head’s forward direction, indicating that learners scanned less of the scene with their eyes alone [77], a narrowing that is a well-established consequence of increased cognitive load [78]. Hesitation, confusion, or simply pausing after deciding to consult the rules could produce similar patterns. Because cognitive and affective states were not measured directly, the mechanism underlying these behavioral changes cannot be determined from the present data.

A further consideration is the substantial heterogeneity in help-seeking behavior across learners. The proportion of trials on which participants sought help ranged from 4.2% to 44.2%, so the most frequent help-seeker consulted the rules roughly ten times as often as the least frequent participant (Section 3.1). Because all participants completed the same task structure, this variation suggests that learner-specific factors contribute to when help is sought. Some learners may seek assistance at relatively low levels of uncertainty, whereas others may persist independently for longer. Prior work has linked such tendencies to learner characteristics including achievement goals and the perceived threat of asking for help [32,33]. Predictive performance also varied considerably between learners. Lift over chance ranged from 1.0 to 5.2 across held-out participants (Figure 7), and the relative pattern across participants was similar across the two model configurations. The clearest example was P18, who sought help on almost half of the trials but for whom the model performed no better than chance. Frequent consultation may be less tightly coupled to a distinct buildup of behavioral change, making upcoming requests harder to distinguish from ordinary task behavior.

This between-learner variation may partly explain the modest performance of the generalized model. Under leave-one-subject-out evaluation, an unseen learner is predicted using patterns learned from other learners, who may differ both in how readily they seek help and in how an emerging need for help is expressed behaviorally. A generalized model may therefore capture only part of an individual’s pattern. Evidence from related domains illustrates the potential cost of this averaging: in emotion recognition from wearable signals, a generalized model reached an $F_{1}$ of 0.43, whereas a personalized model reached 0.92 [79]. Our subject-independent results should therefore be interpreted as a measure of cross-learner generalization rather than as an upper bound on what an individualized system could achieve.

Personalization could be introduced gradually. One option is learner-specific threshold calibration: a lower decision threshold detects more requests but also produces more false alerts, and the appropriate balance may differ between learners. This trade-off is important because unnecessary intervention can interrupt productive struggle [24], whereas failing to provide help when it is needed also carries costs [31]. Threshold calibration could therefore provide a relatively data-efficient first step while preserving the generalized model. As more learner-specific data become available, the model itself could be adapted, which has improved recognition performance with limited per-user data in related work [80]. Where little individual data are available, learners could alternatively be grouped by similarity and initialized from a group-specific rather than fully generic model [81]. Measures of personality or help-seeking attitudes may also help characterize such differences before sufficient behavioral data have accumulated.

Several limitations affect the generalizability of these findings. The first concerns how the onset of help-seeking was identified. Rules were requested by looking at the avatar for 3 s, but this request took no single, clear form. Some participants looked directly and steadily at the avatar, whereas others looked around or glanced at the blackboard, checking whether the rules had appeared, and brief losses of eye tracking occasionally interrupted an otherwise continuous look. Identifying the start and end of each episode therefore required explicit rules to handle this variability, such as merging looks separated by less than 5 s (Section 2.3). Future work would benefit from a help action with a single, clearly defined form, such as a button press or a spoken request, which would reduce the need for such rules and make the onset of help-seeking easier to identify.

A second limitation concerns the scope and structure of the task. Changing the rules without warning was designed to induce uncertainty and reliably elicited help-seeking. This design can produce a recurring sequence in which an unexpected error signals that the rules may have changed and prompts the learner to consult them, which may not fully reflect how uncertainty and help-seeking arise in other learning and training contexts. Each ruleset was learned within a block and then replaced, so knowledge did not accumulate across the session. We therefore could not examine how help-seeking changes as a learner’s understanding develops over a longer procedure, as would typically occur in a training application. Moreover, help took a single form: consulting a fixed reference. Other forms of assistance, such as asking a question, requesting a demonstration, or seeking reassurance, may produce different behavioral signatures. The behavioral changes observed here were also measured during manual assembly, and an emerging need for help may be expressed differently in tasks with other movement demands. Extending the approach to procedural training tasks in which knowledge accumulates and different forms of assistance are available would provide a stronger test of the generality of the observed pattern.

A further limitation concerns the eye-tracking signal. A new gaze value was available on only about half of the logged frames (Section 2.4), which limits the temporal precision with which fixation onsets and offsets could be determined. Fixation measures were therefore compared within participants rather than interpreted in absolute terms.

A final limitation concerns the evaluation. Nested leave-one-subject-out cross-validation, with hyperparameter and threshold selection performed inside the training folds, prevents the held-out participant from influencing these model-selection steps [82]. The window length, prediction horizon, stride and classifier, however, were selected from a sweep evaluated on all participants before the nested procedure, so the reported performance may be optimistic. The nested estimate was similar to the corresponding sweep estimate, but this agreement does not eliminate the possibility of selection optimism because the configuration sweep and nested evaluation used the same participants. Cross-validation also cannot compensate for limited coverage of the learner population, and a larger and more diverse sample would be needed to establish how consistently the behavioral signature generalizes across learners and contexts. We also collected no individual-difference measures, so the substantial variation in help-seeking frequency remains unexplained and the grouping strategy proposed above could not be tested. Although such measures predict help-seeking intentions considerably better than help-seeking behavior [83], they could still provide useful information for future personalization. Finally, blood volume pulse and electrodermal activity were recorded but could not be used because coverage was incomplete across participants. Combining these physiological signals with the behavioral features, when consistently available, is a direct extension of the present work.

## 5. Conclusions

We examined whether an upcoming help request in an immersive VR assembly task can be anticipated from eye, head, and hand movement alone. At the trial level, prediction relied mainly on performance and context features, which summarize completed-trial outcomes or the structure of the experimental design and therefore provide limited temporal resolution for a system intended to act before a learner asks. At the window level, non-verbal behavior alone predicted upcoming requests in learners the model had not seen before, at about 2.5 times the chance level. The turn toward the source of help appeared only in the final moments before a request. Excluding it reduced AUC-ROC only modestly, although fewer requests were detected.

Before a request, hand and head movement slowed, fixations lengthened, and gaze stayed closer to the head’s forward direction. The behaviors the model relied on most, hand movement and eye-in-head eccentricity, departed from ordinary behavior several seconds before the request, whereas the turn toward help appeared only at its end.

These results indicate that help-seeking is partly foreshadowed by changes in behavior that VR systems already record, consistent with the view that the visible request is the endpoint of a longer process. The predictive signal, however, was limited and varied considerably between learners, and it is not yet sufficient for a tutoring system to act on these predictions alone. Determining whether such signals can support proactive intervention will require larger and more varied samples, tasks in which knowledge accumulates and help takes more than one form, a more clearly defined help action, and calibration to the individual learner. 

## Figures and Tables

**Table 1:** Configuration with the highest AUC-ROC and with the highest lift for each classifier across all 480 configurations (LOSO). For every classifier, both occurred at the 3 s horizon. Chance level equals the positive rate.

Classifier	Selected by	*W* (s)	*S* (s)	AUC-PR	Chance	Lift	AUC-ROC
Logistic regression	AUC-ROC	4.5	2.0	0.148	0.061	2.42	0.660
	Lift	3.0	2.0	0.160	0.064	2.50	0.647
Random forest	both	3.0	2.0	0.150	0.064	2.35	0.646
XGBoost	AUC-ROC	3.5	1.5	0.111	0.062	1.78	0.625
	Lift	4.0	2.0	0.132	0.062	2.15	0.600
LightGBM	AUC-ROC	3.5	1.5	0.119	0.062	1.91	0.632
	Lift	4.0	2.0	0.142	0.062	2.30	0.604

**Table 2:** Performance on held-out participants under nested LOSO cross-validation (logistic regression, H=3 s, S=2 s; mean ± SD over 21 participants). Chance level is the mean proportion of positive windows across participants. Lift is mean AUC-PR divided by mean chance level; its range across participants is given in parentheses.

	W=3s	W=4.5s
Windows (positive)	7971 (493)	7701 (450)
AUC-PR	0.159±0.063	0.152±0.082
Chance level	0.064	0.061
Lift	2.49 (1.03–5.23)	2.49 (1.02–5.39)
AUC-ROC	0.667±0.086	0.672±0.100
Balanced accuracy	0.591±0.069	0.576±0.085
$F_{1}$	0.179±0.078	0.153±0.093
Recall	0.399±0.189	0.344±0.211
Precision	0.127±0.070	0.114±0.080

**Table 3:** Performance after excluding windows ending within *g* seconds before a request (logistic regression, C=0.001, L2; mean ± SD over 21 held-out participants). The random control removed the same number of positive windows at random within each participant (20 repetitions); *p* compares AUC-ROC between guard band and control (paired Wilcoxon). At g=0, values differ slightly from Table 2, which used per-fold hyperparameter selection.

	g=0	g=0.6 s	g=1 s
Positive windows removed	0	107	171
Chance level	0.064	0.051	0.043
AUC-ROC	0.673±0.084	0.650±0.083	0.649±0.088
AUC-ROC, random control	–	0.671±0.085	0.668±0.085
*p* (guard vs. control)	–	0.04	0.10
Recall	0.44±0.20	0.25±0.22	0.30±0.22
Precision/chance	2.0	1.7	1.7
Participants AUC-ROC > 0.5	20/21	21/21	20/21

**Table 4:** Features that differed significantly between windows with and without upcoming help-seeking (Benjamini–Hochberg corrected, q<0.05), sorted by effect size within each modality. Yes/No are the medians across participants of each participant’s median feature value; Δ is the median within-participant difference, *r* the matched-pairs rank-biserial correlation, and $n_{\mathrm{dir}}$ the number of participants whose difference was in the direction of the effect. ^*a*^ Count per window; 12 participants had identical medians in both conditions, so *r* and $n_{\mathrm{dir}}$ are based on the remaining 9.

Feature	Yes	No	Δ	*r*	$n_{\mathrm{dir}}$
*Eye*					
Total fixation duration (ms)	1518.3	1394.0	+134.2	+0.78	17
Max. gaze velocity (°/s)	565.7	592.8	−31.8	−0.65	15
SD fixation duration (ms)	154.8	130.5	+17.0	+0.62	15
Mean fixation duration (ms)	262.7	238.6	+8.9	+0.55	14
SD gaze velocity (°/s)	108.6	114.5	−3.4	−0.53	15
Mean gaze velocity (°/s)	69.3	70.7	−4.0	−0.53	15
*Head*					
Yaw direction changes ^*a*^	0.00	1.00	0.00	−1.00	9
Max. speed (m/s)	0.98	1.20	−0.19	−0.97	20
SD acceleration (m/s^2^)	5.55	7.47	−0.86	−0.96	19
Mean speed (m/s)	0.26	0.31	−0.05	−0.94	19
SD speed (m/s)	0.18	0.22	−0.03	−0.94	19
Mean rotation speed (°/s)	37.2	46.0	−5.3	−0.87	19
Mean acceleration (m/s^2^)	3.29	4.57	−0.57	−0.84	18
Pitch range (°)	26.7	30.9	−5.1	−0.80	17
Eye-in-head eccentricity (°)	19.4	20.1	−1.0	−0.80	18
SD rotation speed (°/s)	32.5	38.0	−4.9	−0.70	16
*Hand (active)*					
Mean rotation speed (°/s)	116.1	152.9	−33.3	−0.96	19
Mean speed (m/s)	0.61	0.78	−0.19	−0.95	19
Mean acceleration (m/s^2^)	8.35	10.64	−1.69	−0.93	19
SD speed (m/s)	0.46	0.54	−0.06	−0.91	17
SD rotation speed (°/s)	94.2	111.3	−9.2	−0.91	19
Max. speed (m/s)	2.43	2.68	−0.21	−0.87	19
SD acceleration (m/s^2^)	12.24	14.03	−2.02	−0.78	16
*Hand (supporting)*					
Mean rotation speed (°/s)	49.7	59.9	−10.2	−0.94	19
Mean speed (m/s)	0.27	0.34	−0.07	−0.92	18
Mean acceleration (m/s^2^)	3.35	4.60	−0.95	−0.90	19
Max. speed (m/s)	1.01	1.23	−0.21	−0.89	19
SD speed (m/s)	0.20	0.23	−0.05	−0.83	18
SD acceleration (m/s^2^)	4.93	6.28	−1.13	−0.82	17
SD rotation speed (°/s)	36.2	39.1	−3.9	−0.73	16

## Data Availability

The data presented in this study are available on request from the corresponding author due to privacy and ethical restrictions.

## Appendix A. Feature Definitions

This appendix lists the full set of features extracted from the behavioral streams and the task logs, as described in Section 2.5. Table A1, Table A2 and Table A3 define the 44 non-verbal features (16 eye-tracking, 14 head, and 14 hand features) used in both prediction setups; Table A4 and Table A5 define the performance and context features, which are available in the trial-level setup only.

**Table A1:** Eye-tracking features.

Feature	Description
Fixation count	Number of fixations identified by I-VT within the segment
Total fixation duration	Sum of all fixation durations (ms)
Mean fixation duration	Mean duration of a fixation (ms)
SD of fixation duration	Standard deviation of fixation duration (ms)
Mean gaze velocity	Mean angular gaze velocity between consecutive gaze samples (°/s)
SD of gaze velocity	Standard deviation of angular gaze velocity (°/s)
Maximum gaze velocity	Maximum angular gaze velocity (°/s)
Mean gaze acceleration	Mean angular gaze acceleration (°/s^2^)
SD of gaze acceleration	Standard deviation of angular gaze acceleration (°/s^2^)
Maximum gaze acceleration	Maximum angular gaze acceleration (°/s^2^)
Mean saccade amplitude	Mean angle between the centers of consecutive fixations (°)
SD of saccade amplitude	Standard deviation of saccade amplitude (°)
Maximum saccade amplitude	Maximum saccade amplitude (°)
Gaze switch count	Number of changes in AOI between consecutive fixations
Gaze switch rate	Gaze switches normalized by segment duration (s^−1^)
Gaze transition entropy	Modified gaze transition entropy (mGTE) over fixation AOIs, [0, 1]

**Table A2:** Head movement features.

Feature	Description
Mean head speed	Mean frame-to-frame translational speed of the head (m/s)
Maximum head speed	Maximum translational speed of the head (m/s)
SD of head speed	Standard deviation of head speed (m/s)
Mean head acceleration	Mean absolute rate of change of head speed (m/s^2^)
SD of head acceleration	Standard deviation of head acceleration (m/s^2^)
Head idle fraction	Proportion of segment time with head speed below 0.01 m/s
Mean head rotation speed	Mean angular velocity of head orientation (°/s)
Maximum head rotation speed	Maximum angular velocity of head orientation (°/s)
SD of head rotation speed	Standard deviation of head rotation speed (°/s)
Yaw range	Range of the unwrapped yaw angle (°)
Pitch range	Range of the pitch angle (°)
Yaw direction changes	Number of sign reversals in frame-to-frame yaw change, counting only changes of at least 1°
Mean eye-in-head eccentricity	Mean angle between gaze direction and the head’s forward axis (°)
SD of eye-in-head eccentricity	Standard deviation of eye-in-head eccentricity (°)

**Table A3:** Hand movement features. The active and supporting hand are determined per segment, as defined in Section 2.5.3.

Feature	Description
Mean active hand speed	Mean frame-to-frame translational speed of the active hand (m/s)
Maximum active hand speed	Maximum translational speed of the active hand (m/s)
SD of active hand speed	Standard deviation of active hand speed (m/s)
Mean active hand acceleration	Mean absolute rate of change of active hand speed (m/s^2^)
SD of active hand acceleration	Standard deviation of active hand acceleration (m/s^2^)
Mean active hand rotation speed	Mean angular velocity of active hand orientation (°/s)
SD of active hand rotation speed	Standard deviation of active hand rotation speed (°/s)
Mean supporting hand speed	Mean frame-to-frame translational speed of the supporting hand (m/s)
Maximum supporting hand speed	Maximum translational speed of the supporting hand (m/s)
SD of supporting hand speed	Standard deviation of supporting hand speed (m/s)
Mean supporting hand acceleration	Mean absolute rate of change of supporting hand speed (m/s^2^)
SD of supporting hand acceleration	Standard deviation of supporting hand acceleration (m/s^2^)
Mean supporting hand rotation speed	Mean angular velocity of supporting hand orientation (°/s)
SD of supporting hand rotation speed	Standard deviation of supporting hand rotation speed (°/s)

**Table A4:** Performance features (trial-level only).

Feature	Description
Trial duration	Total trial duration (s), from gear appearance to bin deposit
Error	Binary flag: 1 if any incorrect sort occurred during the trial
Time to first grab	Time (s) from trial start to first grab event
Action count	Total number of grab events during the trial
Board check count	Number of blackboard consultation episodes during the trial
Total board check duration	Total time (s) spent consulting the blackboard
Mean board check duration	Mean duration (s) per consultation episode

**Table A5:** Context features (trial-level only).

Feature	Description
Current block	Ruleset block number (1–4)
Trial number in block	Trial index within the current block
Trial number overall	Global trial index across the full session
New block	Binary flag: 1 if this trial immediately follows a ruleset change
